# canSAR chemistry registration and standardization pipeline

**DOI:** 10.1186/s13321-022-00606-7

**Published:** 2022-05-28

**Authors:** Daniela Dolciami, Eloy Villasclaras-Fernandez, Christos Kannas, Mirco Meniconi, Bissan Al-Lazikani, Albert A. Antolin

**Affiliations:** 1 Department of Data Science, The Institute of Cancer Research, London, SM2 5NG UK; 2 Cancer Research UK Cancer Therapeutics Unit, The Institute of Cancer Research, London, SM2 5NG UK; 3 Molecular AI, Discovery Sciences, R&D, AstraZeneca, Gothenburg, Sweden; 4 MD Anderson Cancer Center, Houston, TX 77054 USA; 5 Present Address: BenevolentAI, London, W1T 5HD UK; 6 Present Address: Dunad therapeutics, Cambridge, UK

**Keywords:** canSAR, KNIME, Standardization, Tautomerism, Compound hierarchy, FDA-approved drugs, Canonicalization

## Abstract

**Background:**

Integration of medicinal chemistry data from numerous public resources is an increasingly important part of academic drug discovery and translational research because it can bring a wealth of important knowledge related to compounds in one place. However, different data sources can report the same or related compounds in various forms (e.g., tautomers, racemates, etc.), thus highlighting the need of organising related compounds in hierarchies that alert the user on important bioactivity data that may be relevant. To generate these compound hierarchies, we have developed and implemented canSARchem, a new compound registration and standardization pipeline as part of the canSAR public knowledgebase. canSARchem builds on previously developed ChEMBL and PubChem pipelines and is developed using KNIME. We describe the pipeline which we make publicly available, and we provide examples on the strengths and limitations of the use of hierarchies for bioactivity data exploration. Finally, we identify canonicalization enrichment in FDA-approved drugs, illustrating the benefits of our approach.

**Results:**

We created a chemical registration and standardization pipeline in KNIME and made it freely available to the research community. The pipeline consists of five steps to register the compounds and create the compounds’ hierarchy: 1. Structure checker, 2. Standardization, 3. Generation of canonical tautomers and representative structures, 4. Salt strip, and 5. Generation of abstract structure to generate the compound hierarchy. Unlike ChEMBL’s RDKit pipeline, we carry out compound canonicalization ahead of getting the parent structure, similar to PubChem’s OpenEye pipeline. canSARchem has a lower rejection rate compared to both PubChem and ChEMBL. We use our pipeline to assess the impact of grouping the compounds in hierarchies for bioactivity data exploration. We find that FDA-approved drugs show statistically significant sensitivity to canonicalization compared to the majority of bioactive compounds which demonstrates the importance of this step.

**Conclusions:**

We use canSARchem to standardize all the compounds uploaded in canSAR (> 3 million) enabling efficient data integration and the rapid identification of alternative compound forms with useful bioactivity data. Comparison with PubChem and ChEMBL pipelines evidenced comparable performances in compound standardization, but only PubChem and canSAR canonicalize tautomers and canSAR has a slightly lower rejection rate. Our results highlight the importance of compound hierarchies for bioactivity data exploration. We make canSARchem available under a Creative Commons Attribution-ShareAlike 4.0 International License (CC BY-SA 4.0) at https://gitlab.icr.ac.uk/cansar-public/compound-registration-pipeline.

**Supplementary Information:**

The online version contains supplementary material available at 10.1186/s13321-022-00606-7.

## Introduction

canSAR (http://cansar.icr.ac.uk) [1] is the largest, public, integrative drug discovery knowledgebase with a primary focus on oncology but also useful and easily extendable to other diseases [2]. canSAR integrates vast multidisciplinary data including chemical, drug, pharmacological, genomic, and protein data with structural biology and protein networks, among others. canSAR is widely used internationally by academia and industry and provides AI-informed target assessment and druggability across the human proteome and support for experimental design in translational research. Crucially, canSAR also provides unique data, curation and annotation, particularly on cancer drugs and their mechanisms of action, and chemical probes in close collaboration with The Chemical Probes Portal [3]. Currently, canSAR integrates information from 3,455,492 unique 2D chemical structures from ChEMBL [4], BindingDB [5], PDB [6], The Chemical Probes Portal [3] as well as additional bioactive compounds that we curated from the literature. In a recent analysis, we identified that many valuable, high-quality chemical probes were originally published outside the medicinal chemistry literature and therefore absent from public databases such as ChEMBL or BindingDB [7]. Accordingly, we started to abstract and curate these valuable chemical structures and associated bioactivity data from the literature in collaboration with The Chemical Probes Portal. During this exercise, we encountered several challenges, particularly: 1. The need to have a robust and transparent chemical registration pipeline to handle these disparate types of data and 2. To handle the different forms and representations of compounds coming from different sources. This latter point is particularly pertinent as compounds can sometimes be registered as separate entities in public databases and linked to different data (e.g., tautomers), and also with different double bond isomerism, salt stripping, stereoisomerism and isotope annotations. To better serve our users, we needed a means to link these compounds and their associated data in a transparent and traceable way; alert about the existence of relevant information that may otherwise be missed and enable the seamless exploration of their bioactivity data. canSAR initially utilised the ChEMBL pipeline because it is transparent, robust and publicly available [8]. However, it was designed to serve a different purpose, namely faithfully preserving the original sources of data, and it did not enable to create the compound hierarchy that we envisioned, including handling issues around tautomers, canonicalization, etc. The NIH/CADD pipeline addressed these issues effectively, but it is built using commercial software and is therefore not available for download and implementation in the public domain [8, 9].

Building on these two approaches, and to facilitate better integration of all these chemical and biological sources of information, we developed a new canSAR chemistry registration and standardization pipeline, canSARchem, and its full open access version, canSARchem_RDKit. Both pipelines are implemented with the KNIME [10] analytics platform, and we have made them freely and publicly available.

## Methods

### Using a non-standard InChI as a unique identifier

To enable this integration, we carefully considered the best standardization and generation of a unique identifier to assign to each chemical structure irrespective of its source. Challenges of large-scale 2D chemical structure database integration related to the use of specific machine-readable chemical structure representations are well documented [11–16]. It is known that widely-used chemical structure representations such as the simplified molecular input line entry system (SMILES) are not unique [17, 18]. The IUPAC developed the Standard InChI (International Chemistry Identifier) to alleviate some of these issues, but certain limitations remain, particularly at handling tautomeric forms, some of which are aggregated into the same Standard InChI [19, 20]. Overall, the choice of the most appropriate chemical structure representation is strategic, also for the assessment of the uniqueness in the dataset itself. Some databases use the Standard InChI as their unique identifier, but it has some limitations, for example at handling some tautomeric forms that are automatically merged [12, 19]. To address this limitation, we decided to use the Non-Standard InChI and the corresponding hashed Non-Standard InChIKey as our uniqueness identifier, which includes the optional fixed-H layer. The fixed-H layer is essential to obtain a different chemical representation for different tautomeric forms, allowing the InChI to be tautomer sensitive [21].

### Developing canSARchem

**Fig. 1:**
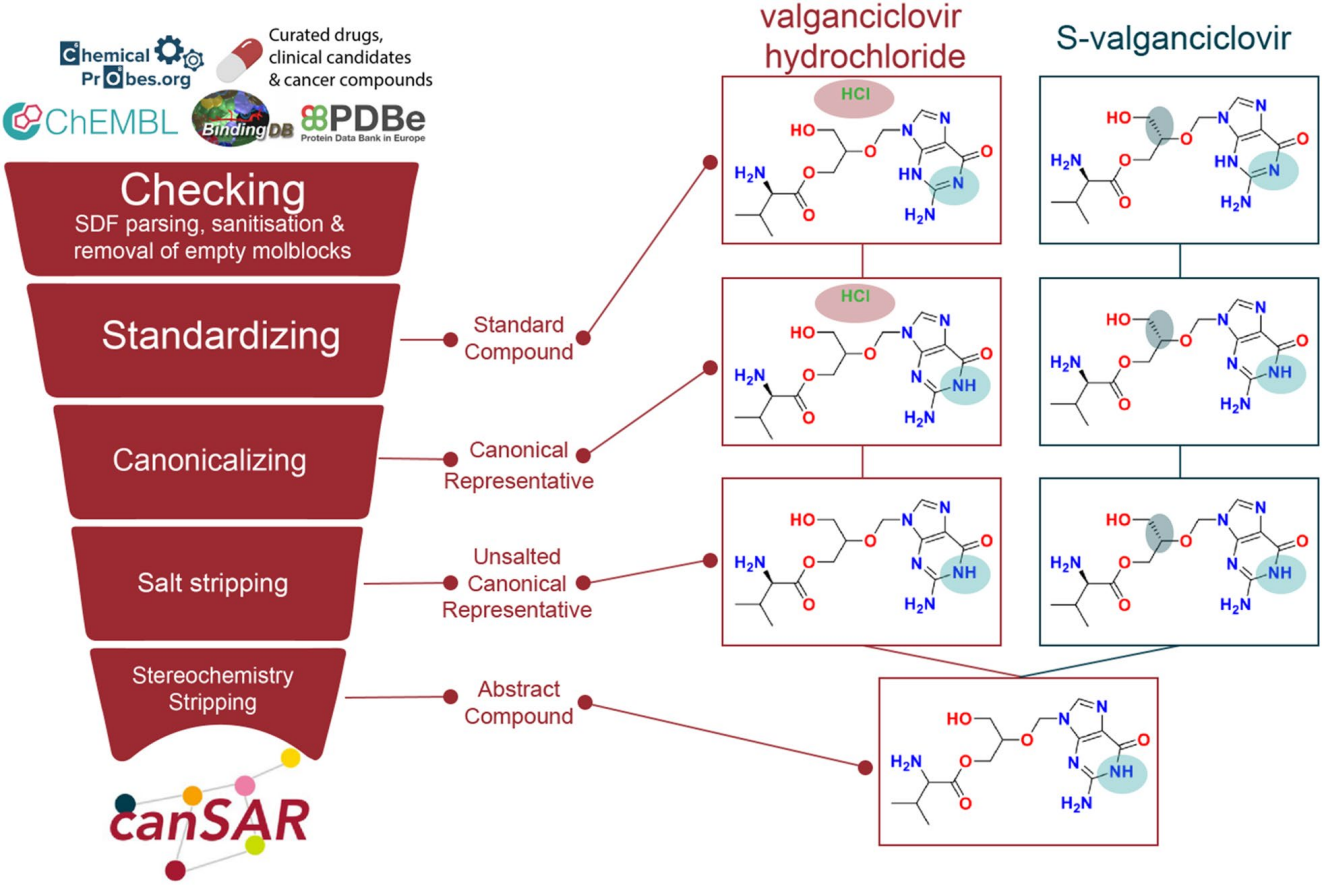
Scheme of canSARchem chemical registration and standardization pipeline. Input chemical structures are firstly validated through the Checking step where SDFs are parsed, molecules with empty mol blocks are removed and valid structures are progressed through a sanitisation step. Standardized compounds are then generated to be submitted to the canonicalization step. Salt stripping with neutralization is used to obtain the unsalted canonical representatives that are finally stripped of stereochemistry and isotopes to give the abstract compounds. In the valganciclovir example, salt and stereochemistry stripping are key steps to enable data integration on the basis of chemical structure. Indeed, the abstract form is the same for the two input structures

canSARchem was developed to be robust, modular and maximally benefit from the user-friendliness and wide adoption of the open-source pipelining software KNIME [22]. In addition, we used the open-source RDKit [23] for specific standardization steps in line with the ChEMBL chemical structure curation pipeline [8]. Automated tautomerisation algorithms are widely known to have limitations; yet enumerating different tautomeric forms of the same compound would be an important tool in grouping related compounds. After extensive assessment, we found that open-source software solutions had significant limitations, discussed below, in managing the tautomerisation step, and identified commercial software that provided a more robust, albeit still imperfect, solution. Therefore, for maximal fidelity to the input structures, we use SDF files as the external chemical data submitted to the canSARchem whenever possible. V2000 molfile format is then used to handle and export registered compounds data to upload in the database. To aid the researcher in identifying relevant biological data from different compounds, we implemented a compound hierarchy to enable the navigation between different forms of compounds (e.g., tautomers, racemic forms, etc.) and their associated bioactivity data. We developed a nomenclature to define each level, building on the definition of NCI/CADD structure identifiers used in PubChem without the generation of the corresponding hash codes, isomeric canonical SMILES or the use of the commercial software [16, 24]. As shown in Fig. 1, after an initial checking, the pipeline standardises the input compounds to give the ‘Standard Compound’ output. Next, canonicalization is performed on the standard compound, to obtain the Canonical Representative. Salts and solvents are then stripped to generate the Unsalted Canonical Representative output before the final step consisting in generating the Abstract Compound devoid of any stereochemistry or isotope labels. The valganciclovir example in Fig. 1 illustrates the use of our pipeline to delineate relationships between compounds using this hierarchy, enabling users to be alerted that (*S*)-valganciclovir is solved in complex, whereas a functional assay is reported against valganciclovir hydrochloride.

### Workflow description and development

The whole process described in this paper is implemented in KNIME (version 4.5.0). The workflow, along with instructions for installation and use, is available on Gitlab at https://gitlab.icr.ac.uk/cansar-public/compound-registration-pipeline. We have also made the workflow publicly available in the KNIME Hub (https://hub.knime.com/danieladolciami/spaces/Public/latest/canSARchem~hquSdFp3di4kiEv). We provide the pipeline openly under a Creative Commons Attribution-ShareAlike 4.0 International License (CC BY-SA 4.0, https://creativecommons.org/licenses/by-sa/4.0/) to ensure maximal utility to the research community.

canSARchem consists of 5 steps:

1. Structure Checker: wrong or problematic structures are discarded.
2. Structures standardization according to MolVS [25] rules as implemented in RDKit Standardized Module (Cleanup function).
3. Generation of canonical representatives, prioritizing aromaticity and protecting tetrahedral stereo centres and double bond isomerism.
4. Salt strip: salts, solvents and fragments are removed.
5. Generation of abstract structure clearing double bond isomerism and sp^3^ stereochemistry along with isotope annotation.

**Fig. 2:**
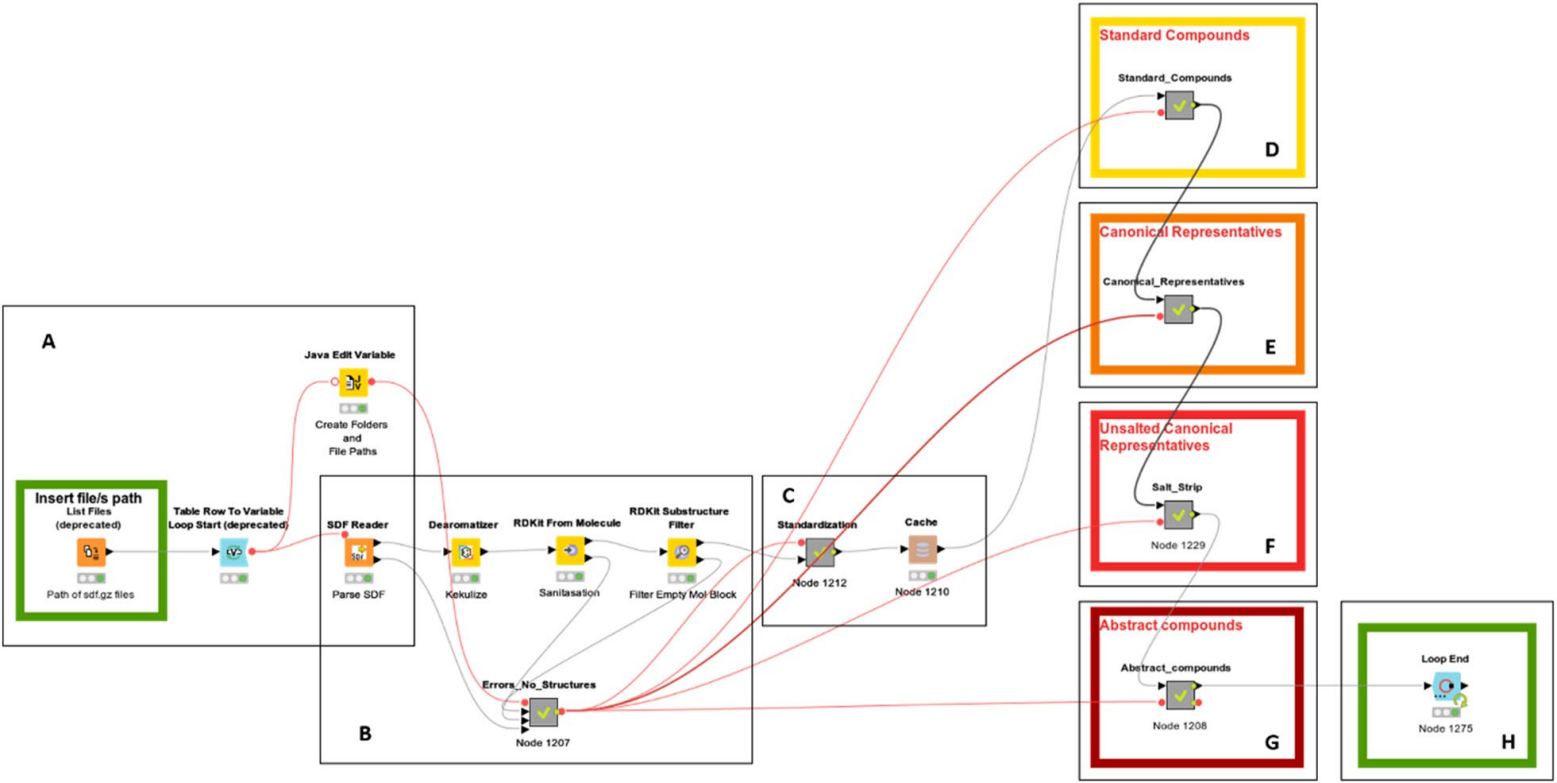
Implemented KNIME workflow for compound standardization and registration. **A** Structure import and creation of variables for the writing of output files. **B** Structures checker. **C** Standardization. **D** Output generation for standardized compounds. **E** Canonical representative generation. **F** Salt stripping and generation of unsalted canonical representative output file/s. **G** Stereoisomery, isomerism and isotopes strip with the generation of the abstract compounds. **H** Node to execute in order to run the pipeline

Figure 2 shows an overview of the workflow. SD files are read and processed in the A block of the workflow. After the rejection of problematic compounds (B), structures are standardized (C) and subsequently exported as Standard Compound output (D). Block E determines the canonical tautomer and generates the Canonical Representative. Block F strips any salt and feeds block G that, after stripping stereochemistry, isomers and isotopes labels generates the Abstract Compound.

All the above steps use KNIME nodes from: Indigo KNIME integration (v2.0.0), RDKit Nodes Features (v202106041552), KNIME Base Chemistry Type and Nodes (v202104131254), ChemAxon/Infocom Marvin and JChem Extensions (v211100), KNIME Python integration (v202106250919), KNIME Java Snippet (v202106241003). RDKit (v2021.09.4) has been used for specific tasks through python scripts.

#### Structure checker

The first step for compound processing consists in importing compounds in KNIME. The *SDF Reader* node in the workflow retrieves the file/s path from the *List files* node. Only files with sdf.gz extension are imported, as specified in the *List files* node. Subsequently, sdf.gz files are processed individually. Output directories and files names are created or edited using the Java Edit Variable node (Additional file 1: Figure S1A).

The *SDF Reader* node imports the correct structures into KNIME to be progressed to the next steps, but, at the same time, unparsed wrong structures go to the second output port and, in turn, are written in the *Errors* folder (with the suffix: *SDF_Parsing_Errors.sdf.gz*, Additional file 1: Figure S1B).

Before converting the SDF structures into RDKit mol format, a Kekulization is performed through the *Dearomatizer* node. This step avoids errors during sanitisation with the *RDKit from Molecule* node concerning how the aromaticity is perceived. This extra step can prevent a large percentage of such errors, enabling one to proceed to the next steps with a higher number of compounds. SDF to RDKit mol conversion is done generating 2D coordinates and with a partial sanitisation of compounds which includes aromaticity checking and correction of stereochemistry. In addition, compounds that cannot be sanitised are written in the *Errors* folder with the suffix: *Sanitisation_Errors.sdf.gz*. In the last step of the checker process, the *RDKit Substructure Filter* node filters out structures with an empty *mol* block and report them in the *No_structures* folder, as described in the Additional file 1: Figure S1B.

#### Standardization

The standardization step is done using the *Cleanup* function of the Standardization module of RDKit (*rdMolStandardize*) that is built on the MolVS standardization tool [25]. During the standardization, functional groups are normalized, separated charges are recombined (e.g., from O^−^–S^2+^–O^−^ to O=S=O), and metal bonds are disconnected. A re-ionization step is performed ensuring the strongest acid ionizes first, charges are neutralized, and stereochemistry information is standardized or removed. A validation step to identify molecules with unusual characteristics is also run to prevent the progression of compounds with issues that cannot be fixed. Before being standardized, compounds are submitted to the *Chunk Loop Start* node, which progresses only 3000 entries for each iteration (note that users can modify this arbitrary number of entries to adapt to the CPUs of the system they are using). In turn, these entries are further split in as many chunks as the available cores, leaving one core free in the *Parallel Chunk Start* node. (Number of available cores is calculated in the *Java Edit Variable* node and treated as a variable). A further kekulization is then performed before the actual standardization that is run through a python script (*Python Script* node). When building this pipeline, we realised that RDKit failed to neutralize some chemical structures. Therefore, we added a *Standardizer* node to neutralize those structures whose charge was not neutralized through the RDKit Standardization (see Additional file 1: Figure S2 for examples). Since this node converts molecules to SDF, a further step to convert again the SDF format into the RDKit mol one is performed. Afterwards, the *Rule-based Row Splitter* node enables the identification of compounds that fail the standardization and will be written to the *Errors* folder with the suffix *_Standardization_Errors*.sdf.gz. Standardized compounds are instead submitted to the *Break Metal Bonds* metanode that can break those metal bonds which are not identified by the RDKit Standardizer tool (see Additional file 1: Figure S3 for examples). After this step, compounds are neutralized. The two following steps are important to generate coordinates and assign stereochemistry preferentially from 3D structure, when available. The two *Python Script* nodes work through RDKit functions (Additional file 1: Figure S4).

The standardized output file/s (Standard Compound) is/are written in the *Results* folder generating Canonical SMILES, InChI, NonStandard InChI and the corresponding InChI keys through the Standard Compound output metanode. All these steps are performed through dedicated RDKit nodes (*RDKit to InChI*, *RDKit Canonical SMILES*). For the generation of the NonStandard InChI, which, importantly, is the uniqueness measure in the canSAR knowledgebase, we used an *RDKit to InChI* node fixing the hydrogen atoms (“/FixedH” has been added on the Advanced tab). All these string representations are written in all the output files (Standard Compound = CH_SC, Canonical Representative = CH_CR, Unsalted Canonical Representative = CH_UCR and Abstract Compound = CH_AC) enabling an easy comparison of the molecules.

#### Tautomerisation and generation of Canonical Representative

The generation of the Canonical Representative has been the most challenging step in the development of the pipeline. Ahead of the canonicalization, we calculate the number of heavy atoms of molecules, through the *RDKit Descriptor Calculation* to prevent the generation of tautomers on very large molecules with more than 100 heavy atoms. Then, canonicalization is performed through the ChemAxon *Tautomers* node protecting charge, aromaticity, chiral centres and double bond isomerism. Although there are well-documented examples of racemization occurring with tautomerization in solution [16], bioactivity data for different enantiomers cannot be assumed to be equivalent and indeed often differ. To ensure fidelity and traceability of data, we prioritise the protection of chiral centres and preserve chirality tags according to the original sources. For use cases where knowledge about different stereoisomers is relevant, we provide a different level of our hierarchy to connect these for the user. The canonicalization step is the most time-consuming of the pipeline with approximately 600,000 molecules processed in 24 h on average [using an Intel(R) Core™ i7-9850H, CPU 2.60 GHz, RAM 32 GB]. Additional extra steps after canonicalization aim at manipulating the output structures through format conversion. Then, in the *Standardize Molecule* metanode, all the string representations needed are generated, as in the case of Standard Compound output (Additional file 1: Figure S5). Despite the charges being protected during canonicalization, we detected a low number of cases (11,661—0.45% of all Standard Compounds) involving salted forms that became charged after canonicalization. As these are artefacts and not related to any biological data, they are rejected from the pipeline. It is worth stressing that the Canonical Representatives may in some cases not be present in any other database and therefore may not be associated with any bioactivity data but they represent a key link to related compounds with potential relevant bioactivities or other metadata and are a necessary step in the creation of the compound hierarchy.

#### Salt strip

Whilst developing the pipeline, we identified that individual nodes were incapable of correctly stripping all the salts. Accordingly, we developed several sequential steps that enable us to correctly strip the salts, addressing the exceptions that we have identified. The first metanode splits compounds into organic and inorganic to avoid stripping salts from the latter ones. Therefore, only organic molecules proceed to the following steps to be stripped from any salts. At this point, only compounds that are formed by more than one molecule are submitted to the stripping process to save time in the calculation. We identified a second exception concerning cases where the salt had a higher number of atoms than the compound of interest. Since most of the salt stripping approaches work by deleting the smallest string, in these specific cases the salt or solvent was kept. To avoid these cases, we used custom lists of solvents and organic and inorganic salts to perform the first strip through the *RDKit Salt Stripper* node. Then, since these lists were not exhaustive for all salts and solvents, (i.e. did not include all the charged forms for inorganic salts such as Bi^3+^), a further step to clear salts has been added employing ChemAxon *Standardizer* node. The *Concatenate* node is then used to concatenate together all the processed molecules to be exported as the Unsalted Canonical Representative output (Additional file 1: Figure S6). In the vast majority of cases, typical bioactivity data reported for compounds grouped at this level of the hierarchy are interchangeable. However, as in any approach, users are encouraged to take care while merging data.

#### Generation of stereo-agnostic, abstract compound

The last step of compounds processing, which we perform through the ChemAxon *Standardizer* node, is the generation of the Abstract Compound. Specifically, stereoisomerism, double bound isomerism (including both cis/trans and E/Z) and isotope annotations are cleared to generate the abstract compound. This is the key structure in the canSAR hierarchy to generate the compound families. The choice of ChemAxon standardizer was made in view of the possibility to clear *E/Z* double bond isomerism. Indeed, most standardizer tools such as the Indigo *Standardizer* node, allow to clear the *cis/trans* but not the *E/Z* isomerism. After standardization, the Abstract Compound output is generated and written in the *Results* folder, as shown in Additional file 1: Figure S7. In the canSAR database, compound uniqueness is assessed through the NonStandard InChI where hydrogens are fixed. Accordingly, compound families are also generated based on the NonStandard InChI of Abstract Compounds and then linked to the corresponding Standardized Compound, Canonical Representative and Unsalted Canonical Representative compounds employing the identifier, that in the case of example files is the chembl_id. Knowledge of different stereoisomers of a compound is useful in many scientific applications, hence we provide it through our hierarchy. However, care must be taken not to automatically combine all data at this level as they may not be equivalent.

### Pre-requisites and instructions to run the KNIME workflow

KNIME is freely downloadable at https://www.knime.com/downloads. The workflow described herein requires a Python 3 environment with RDKit tool installed. A regular license must be obtained from ChemAxon to execute the ChemAxon nodes. Three example input files are also provided as part of the workflow. To process custom input files, the *List Files* node needs to point to the location where the files are stored. *Errors*, *No_Structures* and *Results* directories are automatically created. Output file names are created from the original input file names by adding a suffix. Suffixes provide information on the file content (e.g. *_SDF_Parsing_Errors*.sdf.gz) and the loop iteration (e.g. *_CH_SC_0*.sdf.gz, where “0” indicate the output has been generated during the first iteration). By default settings, the workflow processes 3,000 molecules during each iteration. Four output files (CH_SC, CH_CR, CH_UCR and CH_AC) are generated for each input file and each iteration. As an example to improve clarity, an SD file of 7000 molecules generates 12 output files, all stored in the Results directory.

### Developing canSARchem_RDKit

In order to provide a fully open access version of our pipeline that did not rely on any commercial software to facilitate openness and reusability, we have also developed canSARchem_RDKit. canSARchem_RDKit mirrors the canSARchem pipeline with the only difference that all ChemAxon software has been removed and replaced with RDKit alternatives. The main differences between both pipelines are the following:

1. Standardizer—neutralization of charged compounds following RDKit standardization is performed through the ‘Uncharger’ module of RDKitMolStandardizer instead of using ChemAxon Standardizer.
2. Canonical representative—canonicalization in canSARchem_RDKit is performed with rdMolStandardize.TautomerEnumerator.Canonicalize with the “remove sp3 stereo” and “remove bond stereo” settings set up as ‘False’.
3. Salt strip—RDKit Salt Strip Standardizer (without solvent lists) has replaced ChemAxon Standardizer node in the 4. Salt Strip Standardizer.
4. Isotopes and stereochemistry—the standardizer node from Indigo extension was used to strip isotopes and stereochemistry using standardize-clear-isotope, standardize-clear-cis–trans and standardize-clear-stereo. However, the node has no option to strip E/Z isomerism. Accordingly, we added a string manipulation node to strip E/Z isomerism removing slashes or back-slashes from SMILES.

canSARchem_RDKit is available in the same Gitlab repository as canSARchem, and we have also deposited it in the KNIME Hub (https://hub.knime.com/danieladolciami/spaces/Public/latest/canSARchem_RDKit~7Qe3vnQZ9zpIla4Z). The differences between canSARchem and canSARchem_RDKit have been further tested with a list of 5735 compounds and the results are presented in the results and discussion section.

## Results and discussion

### Integration of chemical and pharmacological data

We created a new chemical registration and standardization pipeline, canSARchem. Compounds processed through the pipeline can be grouped according to their Canonical Representative, Unsalted Canonical Representative and Abstract Compound, regardless of their original representation. Importantly, we processed all compounds in the public canSAR database with this new pipeline, enabling users to rapidly identify alternative representations of a chemical compound and explore their associated bioactivities where appropriate. Indeed, while we keep the bioactivity data registered versus the original compound form, the user is alerted of the presence of related compounds that may hold useful information.

To this aim, five sequential steps are carried out on input molecules imported from external databases such as ChEMBL, BindingDB, PDB or our unique curated data. As shown in Table 1, the Standard Compound output has been generated through the structure checker and standardization and then submitted to canonicalization to give the Canonical Representative output. Accordingly, Standard Compounds and Canonical Representatives enable to group together compounds with the same Canonical Representative, regardless of their input structure. The Canonical Representative output is then used to generate the free base Canonical Representatives by stripping salts, solvents and small fragments to give the Unsalted Canonical Representative output. Finally, the Abstract Compound is obtained clearing stereochemistry, cis/trans and E/Z isomerism and isotopes annotations.

**Table 1:** Description of canSAR hierarchy and steps to generate each output

3,157,884 unique 2D structures in canSAR	canSAR Hierarchy	Input file	Description	Steps
2,668,609 Standard Forms and Canonical Representatives	Standardized Compound (SC)	Original source DBs in SDF format	Standard form	1. Checker and 2. Standardizer
				SDF parsing and filtering of empty molblocks
				RDKit sanitization
				Structure Standardization through RDKit Standardizer
	Canonical Representative (CR)	SC Output	Canonical Representative	3. Generation of Canonical Representative
				Generation of at most 30 canonical tautomers
				Prevent canonicalization in the presence of chiral center
				Time-out for canonicalization set at 250 ms
2,304,805 Unsalted Canonical Representatives	Unsalted Canonical Representative (UCR)	CR Output	Free base canonical tautomer	4. Salt strip
				Strip inorganic and organic counterions
				Strip solvents and fragments
				Strip shorter SMILES string
				Keep first fragment with two identical SMILES strings
				Neutralization
2,162,736 Abstract Representation	Abstract Representation (AR)	UCR Output	Abstract representation (Canonical compound stripped of salts and stereochemistry)	5. Generation of abstract structure to get parent compounds
				Strip stereochemistry
				Strip cis/trans and E/Z isomerism
				Strip isotopes

The total number of unique 2D structures in canSAR is reported for each hierarchy level together with the input file and the steps carried out to generate the corresponding output files

In canSAR there are, in total, 2,668,609 unique Standard Compounds and Canonical Representatives, 2,304,805 unique free base/acid Unsalted Canonical Representatives and 2,162,736 Abstract Compounds, encompassing clinical candidates, research compounds and chemical probes. Importantly, the 17.6% drop of structures from Standard Compounds and Canonical Representatives to Abstract Compounds confirms the importance of the hierarchy generation for data aggregation. It is important to stress that data associated with one form of the compound is not necessarily immediately transferable to other forms. For example, the protein bioactivities of two stereoisomers are not necessarily the same. Meanwhile, some biological data may be artificially disconnected because of differences in the tautomeric registration of a compound at the source. But it is always important for the researcher to know what data are available in a fully transparent and traceable way. Bioactivity data or crystal structures registered for different forms of a compound will be useful and will be highlighted in the canSAR interface.

**Fig. 3:**
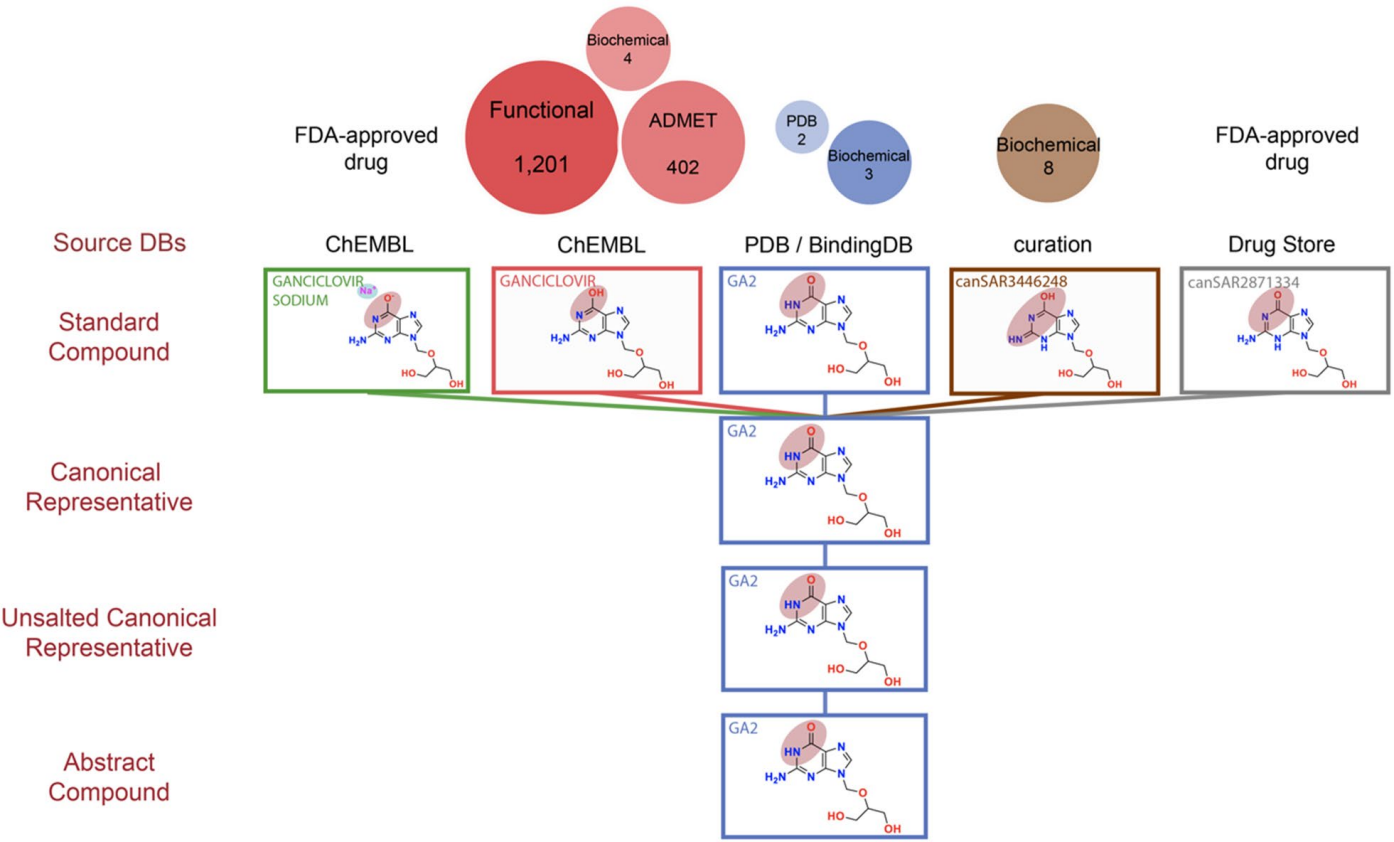
Example of the advantages of canSAR hierarchy. Different tautomers, salt forms and double bond isomerism for ganciclovir are submitted to canSARchem. The generation of canonical representatives, unsalted canonical representatives and abstract compound allows to group and consolidate all the 5 input entries in the same family enabling the user to quickly identify all the alternative forms that exist in canSAR. This, in turn, makes the user aware of the data measured and registered versus the different compound forms. Circles at the top represent the different data types and the number of bioactivity data points associated with each one of them. As it can be observed, the biochemical assays of ganciclovir against metabotropic glutamate receptors from BindingDB (associated with GA2, in blue), could be missed if the user only explored the tautomer associated with the ‘GANCICLOVIR’ name tag

**Fig. 4:**
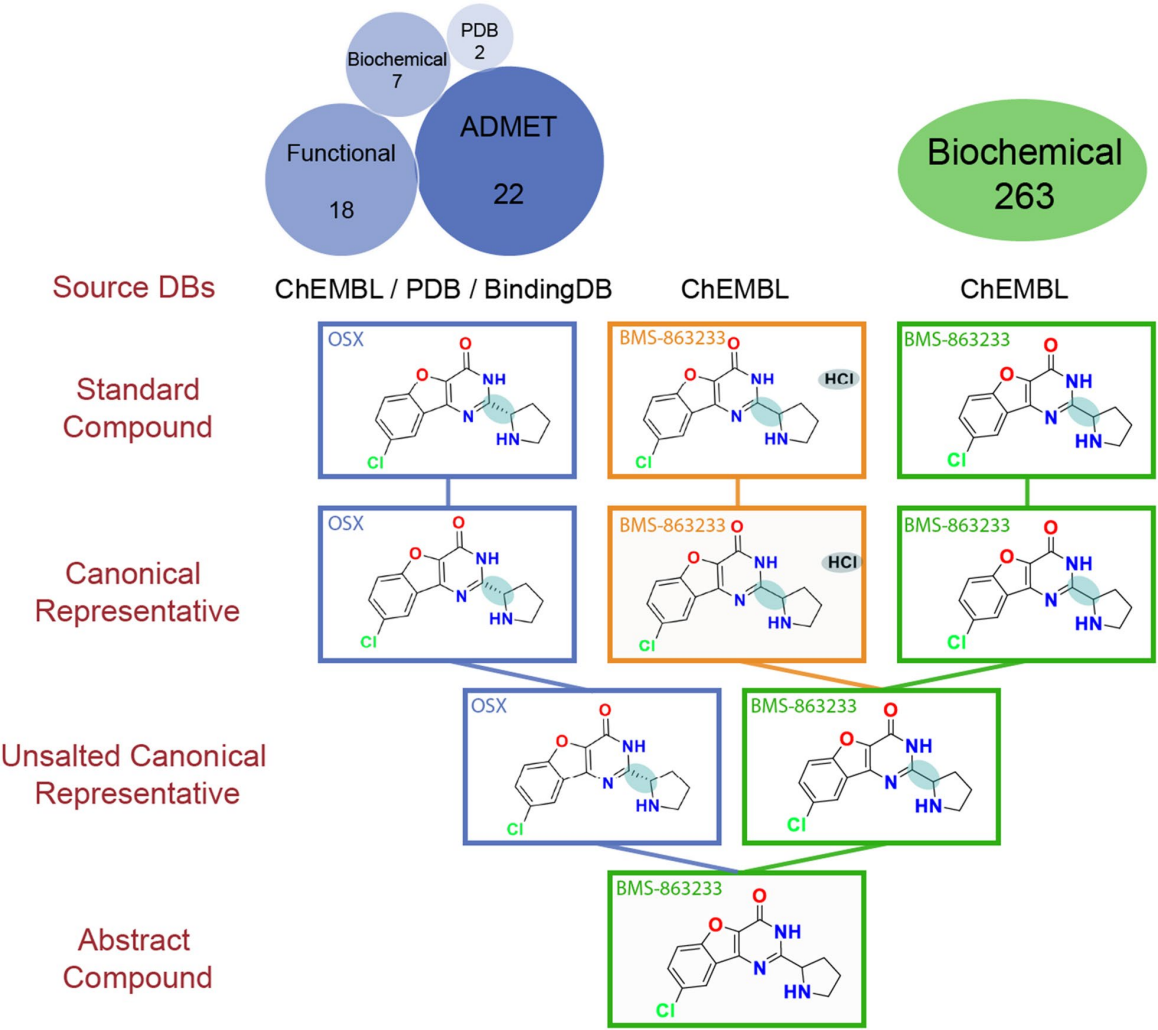
Example of the advantages of canSAR hierarchy (II). Different salt forms and stereoisomers for BMS-863233. Salt strip and clear stereochemistry steps allow to generate the compound family. It is important to highlight that the racemate BMS-863233 is associated with a large-scale selectivity profiling data whilst the pure enantiomer OSX is associated with structural and ADME data. Circles at the bottom represent the different data types and the number of bioactivity data points associated with each one of them

To illustrate the utility of the canSARchem pipeline, we report two examples in Figs. 3 and  4 that demonstrate the challenges in data aggregation from several resources and how our hierarchy can help the user identify potentially valuable data. In the case of Ganciclovir (Fig. 3), five different input structures with different tautomeric and salt forms were submitted to the canSAR pipeline from different source databases (ChEMBL, BindingDB, PDBe, raw curation and Drug Store). The generation of the Canonical Representative enabled to group all these forms into one family. As shown in the top part of the image, the data registered versus each compound form is significantly different. It is particularly important to highlight that there is one unsalted tautomeric form registered in ChEMBL (GANCICLOVIR, in red) whilst a different one is registered in the PDB/BindingDB (GA2, in blue) that have significantly different data. For example, BindingDB reports binding affinity for Ganciclovir against metabotropic glutamate receptors from a patent application that could be missed if only the other tautomer was explored. Moreover, a recent paper that was abstracted in canSAR reported binding affinity data for another tautomer of Ganciclovir (canSAR3446248) against the NUDT15 hydrolase, whilst the old Drug Store database had registered yet another Ganciclovir tautomer. The canSARchem hierarchy enables to alert the user that these alternative compound forms exist to facilitate the exploration of their bioactivity data. The compound synopsis in the canSAR website has been particularly designed to enable this by raising awareness of these alternative forms of compounds in the same hierarchy and associated data (https://cansarblack.icr.ac.uk/compound/1070449/synopsis/structure).

Another example of the benefit of the canSAR hierarchy is BMS-863233 (Fig. 4). In this case, the PDB, ADME and functional data are registered versus 0SX, which has a defined stereochemistry; but the ‘BMS-863233’ name, clinical candidate tag and large-scale kinome profiling data are registered against the racemate form. This is somewhat surprising as both large-scale profiling data, co-crystallization and ADME are typically measured against the same compound form—the one in clinical trials. In general, the data associated with an enantiomeric form should not be merged with the racemate as both can have different properties. However, a close inspection at the original publication shows that the clinical candidate is enantiomerically pure and thus the name tag and clinical candidate tag should be associated with 0SX and not with the racemate. It is also very likely that the large-scale kinome profiling was also performed on the enantiomerically pure compound, but the stereochemistry was not appropriately reported in that publication and thus the data ended up associated with the racemate. Therefore, exceptionally, in this particular case, users may want to consider merging the bioactivity data of the racemate and enantiomerically pure compounds. This example illustrates the complexities of integrating chemical and bioactivity data, the existence of errors in public databases that need to be taken into consideration, and how the canSAR pipeline can easily flag the existence of alternative forms of compounds and associated data that may need to be considered by the users.

### Challenges of implementing the chemical standardization pipeline: Canonical Representative generation

As expected, the most challenging step in the implementation of this pipeline is the tautomerisation as part of the generation of the Canonical Representative. Although canonicalization is widely recognised as a challenging, time-consuming step and avoided in many databases [8], 67% of chemical compounds are estimated to be affected by tautomerism [16]. Therefore, we felt it was important to address this issue to our best ability and introduce a canonicalization step to link compounds into families and alert on associated data as best as possible. Various algorithms for tautomer generation and enumeration are available but finding the best tool is challenging as all approaches are known to have limitations [16, 26–28]. We, accordingly, explored four different approaches, namely *Ambit’s Tautomer Generator* node from Knime-CDK extension, *MolVS Tautomer canonicalization* [29], the canonicalization function from *RDKit Tautomer enumerator* [30] and *Tautomers* node from ChemAxon/Infocom JChem extensions. Unfortunately, *Ambit’s Tautomer generator* node has no option for the generation of the canonical tautomer and is consequently not a valid option. *MolVS Tautomer canonicalization* does not allow stereochemistry protection when the proton shift involves a chiral carbon with the consequent loss of chirality annotation producing misleading results in our compound hierarchy. This issue has been partially fixed in the very recent release of *RDKit Tautomer enumerator*. Therefore, we have performed a thorough comparison of ChemAxon and RDKit in tautomer canonicalization in terms of both the tautomeric form obtained and execution time on the three example files provided in the KNIME workflow released with this paper. First, we generated all possible tautomeric forms for the 5,735 unique compounds in the example files using *Ambit’s Tautomer Generator* node. This step generated 8,706 unique molecules that were submitted to canonicalization protecting sp^3^ stereoisomerism and double bond isomerism with the two approaches. The results showed that RDKit was less effective at protecting stereoisomerism and double-bond isomerism in comparison to the more conservative approach used by ChemAxon. Indeed, 2019 molecules were modified during the canonicalization with RDKit, with 640 new generated structures, whilst ChemAxon modified 1826 compounds with only 298 new structures. In most cases, the higher number of molecules modified by RDKit is due to double bond protection that is not behaving as expected, as illustrated in Additional file 1: Table S1.

In terms of execution time, RDKit is faster than ChemAxon as the whole workflow runs in about 5 min with the *Python Script* node for *RDKit canonicalizer* whereas it needs approximately 10 min to process the example files with ChemAxon *Tautomers* node. However, if the approach was used in KNIME through a *Python script* node and running on a bigger number of compounds, the job would likely be halted as we have experienced generating canonical tautomers through *Python script* nodes on bigger databases. Therefore, the speed advantage of RDKit might not be an advantage in KNIME.

**Fig. 5:**
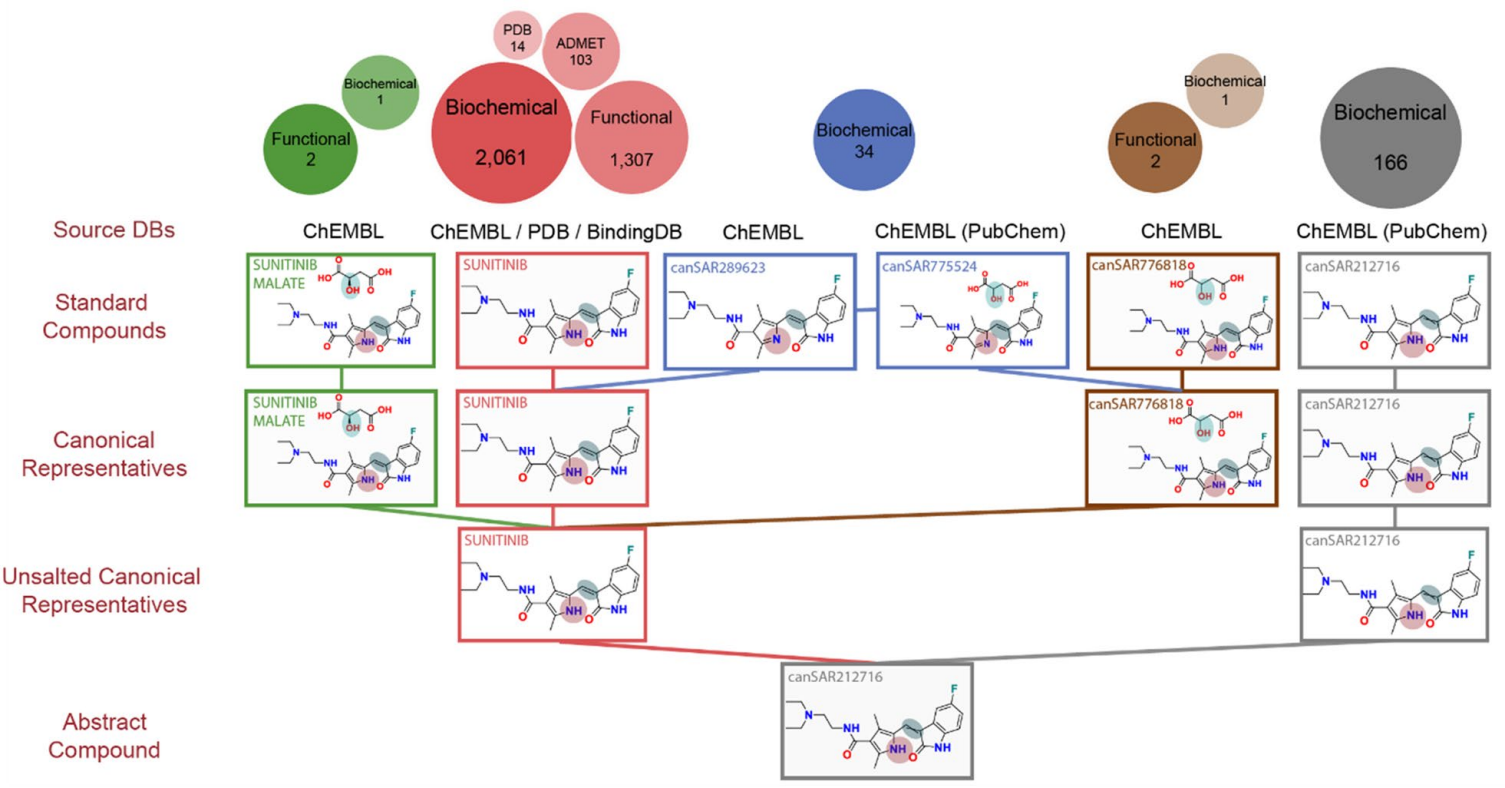
Hierarchy of sunitinib. Example of the limitations of canSARchem hierarchy. Different tautomers, salt forms and double bond isomerism for sunitinib are submitted to canSARchem. The generation of canonical representative, unsalted canonical representative and abstract compound allows to group and consolidate all the 6 input entries in the same family. However, the 3*H*-pyrrole of canSAR289623 and canSAR775524 (in blue) has very different properties than the pyrrole of the other compounds in the family and a high energy barrier of interconversion that is unlikely to enable tautomerism between these compounds in solution. Therefore, it is debatable if these compounds belong to the same hierarchy. Circles at the top represent the different data types and the number of bioactivity data points associated with each one of them

Overall and despite our efforts to use open software wherever possible, we find that ChemAxon *Tautomers* node outperforms the other approaches we tested. The criteria we used were focused on protecting double-bond isomerism and stereoisomerism. While errors were still observed and limitations identified, it was the method that provided the most robust outputs and consequently is our choice for canonicalization. Although successful in most cases, we have identified edge examples where the ChemAxon tool incorrectly standardised compounds. An interesting example to consider is that of sunitinib (Fig. 5). ChemAxon identifies canSAR557724 as a tautomer of sunitinib, although the sp^2^/sp^3^ transition is highly unlikely. This exemplifies a potential limitation of our pipeline because the inclusion of canSAR557724 and canSAR289623 in the same compound hierarchy as sunitinib could be debatable. It is important to highlight that all methods need improvement to better handle tautomers. The availability of more robust open-source tautomerisation and canonicalization pipelines will be transformative for the in-silico chemistry community. Despite the limitations, in order to provide a fully open access pipeline to the community we have also developed canSARchem_RDKit, that does not use ChemAxon software. We have compared canSARchem and canSARchem_RDKit with a list of 5737 compounds. A different output on the standardizer step (see “Methods” section) was found in only one compound (CHEMBL3740280). As expected, the major differences between both pipelines originated in the Canonicalization step, where 750 molecules had a different output compared with the canSARchem pipeline that uses ChemAxon. In addition, 1015 molecules had a different output after salt strip and 763 molecules were processed differently after stripping isotope and stereochemistry information. Despite these differences finally translated into only one additional family, the differences in family numbers will likely increase significantly when large databases are processed. Users should be mindful of the differences between both pipelines and their strengths and limitations.

### Standardization success and modification rates

**Fig. 6:**
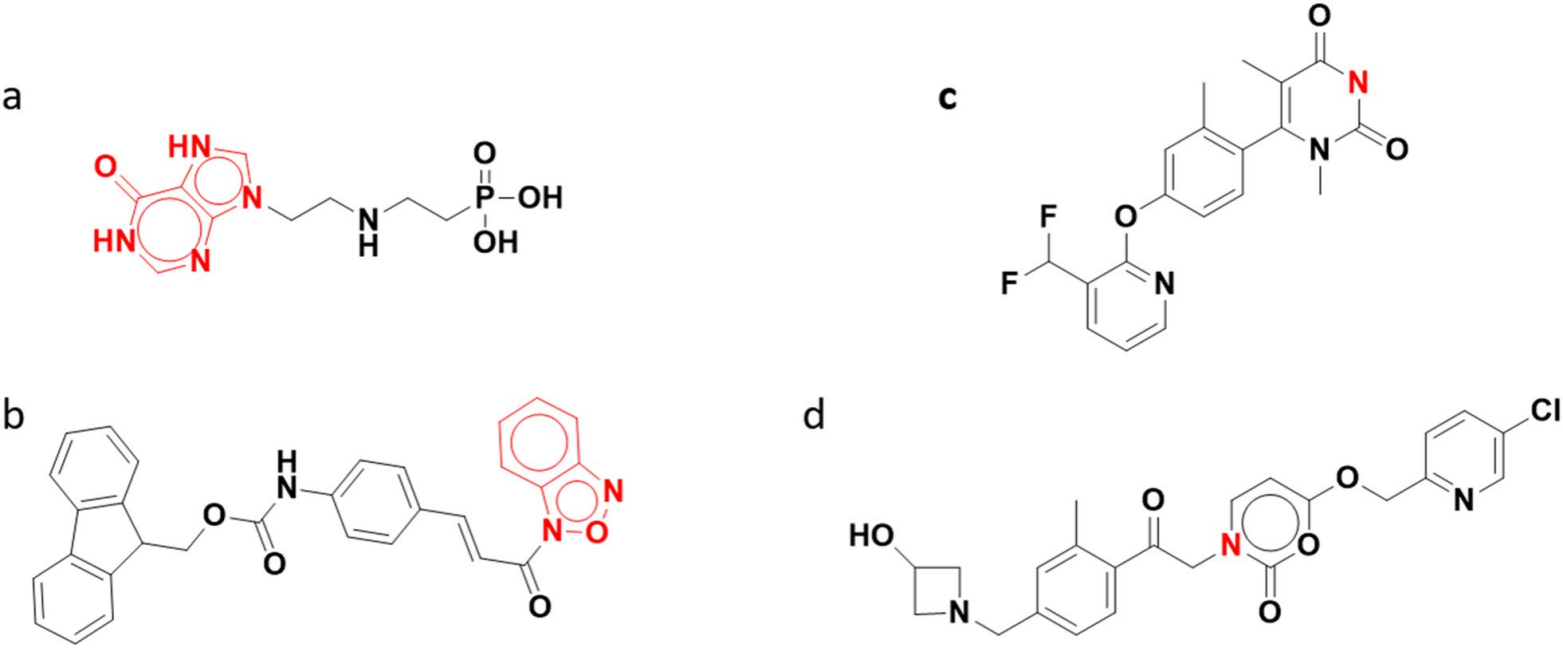
Examples of rejected structures for aromaticity (**a** and **b**) or valence (**c** and **d**) errors. **a** BindingDB MonomerID 60884 and **b** BindingDB MonomerID 185783 are incorrectly represented as aromatic, wrong portion of the molecule are highlighted in red. In **c** BindingDB MonomerID 142162 and **d** BindingDB MonomerID 289106, the valence of the red Nitrogen atom is wrong

In order to standardize compounds and create a compound hierarchy that enables grouping molecules into families, we processed all the canSAR data sources, both external (ChEMBL, BindingDB and PDB) and internally curated through canSARchem. This accounts for a total of 3 million compounds. The overall processing success rate was 97.53% (Table 2). In total, 70 k molecules failed, with 99.4% of them imported from BindingDB (using BindingDB download file named Terse_m11_2020). The most common problems of rejected compounds were related to aromaticity and valence (Fig. 6). Examples of aromaticity errors are presented in Fig. 6a and b, whereas Fig. 6c and d represent structures with malformed valence for structures from BindingDB (Structure checker, Step 1, Table 1).

**Table 2:** Number of compounds from external sources processed through the canSAR pipeline

		ChEMBL27	BindingDB Terse_m11_2020	PDB
	# Structures in source db	1,941,411	898,561	35,258
1.Checker	SDF parsing errors	0	3	0
	RDKIT sanitization errors	1	70,385	0
	Empty molblock	0	6	0
2. Standardizer	Total standardized structures	1,941,410	828,167	35,076
3. Canonical Tautomer generation	Modified structures	179,276 (9.23%)	19,721 (6.14%)	2911 (8.3%)
	Total new structures	101,642	1207	2862
4. Salt strip	Structures strip from salt, fragments, solvents	101,833 (5.2%)	1264 (0.39%)	266 (0.75%)
5. Abstract structure generation	Generated abstract structures	682,321 (35%)	118,074 (36.78%)	19,691 (55.85%)

Compounds modified in each pipeline step are reported for every external database

All checked molecules successfully passed the standardization step (step 2, Table 1), where functional groups are normalized, metal atoms bonds are broken, charges are neutralized, as described in the Methods section. In the subsequent step of tautomerisation, 201,908 total structures (7%) were modified to the Canonical Representative, with 105,902 new molecules generated (3.7%). Of note, the modification rate for the PDB database (8.3%), ChEMBL (9.23%) and BindingDB (6.14%) were similar (Table 2). As a result of the canSARchem pipeline, the bioactivity data corresponding to different tautomeric and other forms can now be easily explored and consolidated. For example, in Fig. 3 the binding data of ganciclovir against metabotropic glutamate receptors from BindingDB (associated with GA2), which could be missed if you only explored the tautomer associated with the ‘GANCICLOVIR’ name tag, can be quickly identified via the compound family. Other examples include tivozanib and its tautomer AV9, and pemetrexed and LYA, all cases, where the PDB structures are registered versus a different tautomeric form than the rest of bioactivity data, and thus exploring only the structure associated with the drug name may lead to the user missing very valuable crystallographic information that is associated with a different tautomeric form. Other cases that show the value of the canSARchem hierarchy are examples of two different tautomers co-crystallized against different entries in the PDB. For example, 2-(4-Hydroxyphenylazo)benzoic acid (HAB) is co-crystallized against Streptavidin from *Streptomyces avidinii* (PDB ID: 1SRE) whilst its tautomer 55R is co-crystallized against Avidin from *Gallus gallus* (PDB ID: 5CHK). Both compounds are brought together in the canSAR hierarchy, making it easy for the user to recognize that an alternative form is co-crystallized against another protein, when one of the compound forms is searched which provides useful information (https://cansarblack-alpha.icr.ac.uk/compound/2871690/synopsis/structure).

It is worth highlighting that we submitted to canonicalization only compounds with a number of heavy atoms lower than 100, in order to prevent the pipeline from halting. As a result, 131,616 molecules (35,101 from BindingDB, 95,393 from ChEMBL and 1122 from PDB) were not submitted to the canonicalization step. Therefore, the numbers reported above may underestimate the number of molecules that were already in the canonical form or do not have tautomers and have not been modified. Moreover, the protection of stereochemistry and double-bond isomerism during canonicalization could have also contributed to an underestimated modification rate. For example, 36% of substances were altered by canonicalization in the Standardize Valence Bond step in PubChem database [9]. However, other studies show a different modification rate, with tautomerism demonstrated to be possible for around 67% of chemical structures [16]. The accurate estimation of the modification rate is also dependent on how the number of canonicalized structures is computed. Herein, we were more interested in counting molecules that differ in their Standard Compound and Canonical Representative levels, to understand how much the canonicalization step affected our final aim of linking associated biological data. In this respect, the percentage of newly generated structures over the modified ones (52.4%) indicates that 47.6% of generated tautomers match with at least one existing structure, that was in the canonical form. This highlights the value of canonicalization to enable chemical structure-based data aggregation regardless of the input tautomeric form, despite the limitations of current canonicalization algorithms that we have already discussed.

The Canonical Representatives were then submitted to the salt strip step where salts, solvents or fragments are removed (Step 4, Table 1). 103,284‬ molecules (3.69%) were affected by salt strip, generating the Unsalted Canonical Representative output.

In the last step (step 5, Table 1) for Abstract Compound generation, 807,487 (28.84%) were modified, enabling grouping compounds in families. Abstract Compounds have been generated clearing tetrahedral configuration, *cis/trans* and *E/Z* isomerism, and isotopes.

### Comparison with published standardization pipelines

In order to assess the global performance of canSARchem, we performed a comparative analysis with PubChem [9] and ChEMBL [8] pipelines. The comparison involved three main aspects: i. Most relevant features and differences among the three pipelines, ii. The impact of these different approaches on the output standardized compounds and iii. Differences in detection and rejection of problematic structures.

Table 3 summarises the comparison of the most relevant features of the three pipelines, which are all publicly accessible. canSARchem is freely available as a KNIME workflow, with a license required for ChemAxon/InfoCom KNIME nodes. It’s worth noting that for academic users, ChemAxon provides a freeweb license to perform the canonicalization step but the corresponding KNIME node is not freely available. Although we made our best effort to use open-access tools for canSARchem, ChemAxon suite outperforms other open-access KNIME-accessible packages for canonicalization. The overall advantages of the KNIME workflow format consist in the integration of an easy to use software (without knowledge of coding) with real-time visualization of executed nodes enabling inspection of the output of each step facilitating troubleshooting as well as modifications. Moreover, since we provide canSARchem under a CC BY-SA license, users can easily modify any of the steps in the pipeline to include different tautomerization modules or reconfigure any KNIME node, but of course, this will impact the final result.

**Table 3:** Comparison of ChEMBL, PubChem and canSAR chemical structure standardization pipelines

		canSAR pipeline	ChEMBL pipeline [8]	PubChem pipeline [9]
	Pipeline availability	Freely available as a Knime Workflow (ChemAxon components require a license)	Open source, public available in GitHub and as Conda package	Open source web-based and programmatic interface
Chemical Structure Curation	1. Checker	**✔**	**✔**	**✔**
	2. Standardizer			
	2.1 Aromaticity standardization	Kekulization	Kekulization	Kekulization
	2.2 Atom valence	**✔**	**✔**	**✔**
	2.3 Radicals	**✔**	**✔**	**✔**
	2.5 Hydrogens treatment	Remove explicit Hs	Remove explicit Hs	Convert implicit Hs into explicit
	2.6 Metal bonds disconnection	**✔**	**✔**	**✔**
	2.7 Apply normalization rules	**✔**	**✔**	Conversion of functional group into the preferred form
	2.8 Verify stereochemistry	**✔**	**✔**	**✔**
	3. Generation of Canonical Tautomers	**✔**	**✘**	**✔**
	4. Salt Strip	**✔**	**✔**	**✘**^a^
	5. Abstract structure			
	5.1 Tautomers	**✔**	**✘**	**✘**^a^
	5.2 Stereoisomers	**✔**	**✘**	**✘**^a^
	5.3 E/Z and cis/trans isomers	**✔**	**✘**	**✘**^a^
	5.4 Isotopes	**✔**	**✔**	**✘**^a^
	5.5 Salts, solvents	**✔**	**✔**	**✘**^a^
	Uniqueness identifier	NonStandard InChI	Standard InChI and corresponding hashed InChIKey	De-aromatized isomeric canonical SMILES
	Actions on compounds which fail to be processed	Compounds are corrected where possible Compounds failed to be sanitised or standardized are not loaded into the DB	Different actions based on perceived errors Compounds with fatal errors are not uploaded into ChEMBL database (penalty score of 7) or uploaded without Molfile (penalty score of 6) or uploaded but prioritized for manual curation (penalty score 5 or 2)	Structures rejected from the standardization pipeline are not pushed into the Compounds database
	Employed tools/libraries	RDKit, MolVS and ChemAxon	RDKit and MolVS	OpenEye Scientific Software, Inc. C^++^
	Parent compounds usage	Canonical tautomers as well as compounds strip from salt and chirality are used to create a compound hierarchy displayed in the structure synopsis	Compounds strip from salts and isotopes are used to get parents shown as alternative forms of compounds	Canonicial tautomers, isolated and neutralized covalent units used to generate related, parent and component compounds
	Mapping bioactivity data	Bioactivity and structural data on the same compound form are aggregated through chemical structure Bioactivity data are mapped against the form it is measured on	Bioactivity data on the same compound form are aggregated through chemical structure Bioactivity data are mapped against the form it is measured on	Bioactivity data are linked to unstandardized compounds (substances)

Differences and similarities of the various step for compounds standardization are highlighted

^a^Not performed through the released pipeline. For compounds registered in DB by the PubChem team, parent structures can be retrieved

ChEMBL pipeline is available in GitHub and as Conda package. PubChem can be accessed through open-source web-based and programmatic interfaces. In the case of PubChem, only the generation of canonical tautomer is possible through the web-based application, but not the salt strip or the generation of the abstract structure that however is retrievable for compounds registered in the PubChem DB (steps marked by a superindex ‘a’ in Table 3). For the purpose of this paper, steps not typically available to users through the available tool or not described in the PubChem chemical structure standardization paper are not discussed. The main difference for the standardization step is the conversion of Hydrogens from implicit to explicit operated by PubChem pipeline that differs from canSAR and ChEMBL approaches. Of note, canonicalization is not performed by the ChEMBL pipeline as a result of their decision to avoid the generation of tautomers [8]. The abstract structures in ChEMBL are obtained through the GetParent component and consist of salt strip and removal of information about isotopes. In canSAR, we decided to strip stereoisomerism and double bond isomerism instead, in order to generate inclusive compounds family with a higher probability to connect data registered against different compound forms. Other important differences concern the uniqueness identifier and the actions on compounds that fail to be standardized. In ChEMBL, InChI and the corresponding InChIKey have been chosen as uniqueness identifiers. PubChem team opted for de-aromatized isomeric canonical SMILES to define a structure as unique. Since one of the risks of using SMILES is the generation of the string itself, in canSAR, we chose Non Standard InChI with fixed Hydrogens as our uniqueness identifier. Indeed, the FixedH option allows appending an extra layer to the InChI string which is essential for making it specific for a single tautomeric form of the structure [21]. Checker and standardization steps are generally used as a choice to reject/accept compounds. However, canSAR approach differs from ChEMBL and PubChem. Using RDKit sanitisation, canSAR pipeline attempts to correct kekulized forms and stereochemistry where possible. Compounds that fail steps 1 and 2 are not imported into the canSAR database. The ChEMBL ‘checker’ component scores compounds before eventually loading them into the database, with the action undertaken depending on the score itself. The PubChem pipeline, in contrast, does not push any rejected structures into the database. Both canSARchem and ChEMBL pipelines are developed on RDKit and MolVS tools. However, ChemAxon suite is used in canSARchem to generate canonical tautomers, for salt stripping and generation of the abstract structure. PubChem chemical registration pipeline was built using the commercial software OpenEye. Parent structures are used to link alternative forms of compounds. The same approach of registering bioactivity data versus the compound form it is measured on is adopted by all three databases.

Regarding the second aspect of the comparison, that is the output compounds generated, Table 4 illustrates with examples how structures are processed by the three pipelines for standardization. As mentioned previously, in PubChem, the generation of the parent structure is unavailable in the web-based tool and therefore has not been considered. The three pipelines similarly fix the hypervalent Nitro group in entry 1 as well as disconnect the metal bond in entry 2. Of note, canSARchem generated a different parent structure compared to ChEMBL. Indeed, Na^+^ is stripped in the salt strip step (to get the Unsalted Canonical Representative output) in canSARchem and the following neutralization generate benzoic acid as a parent structure (Abstract Compound). Example 3 shows how ChEMBL standardizes sulphoxide with charge-separated forms, at odds with PubChem and canSAR. Standardization of doxycycline (entry 4) resulted in three different compound forms. The ChEMBL pipeline does not modify the input structure, due to lack of tautomer generation, whereas two different outputs are obtained through PubChem and canSARchem pipelines corresponding to different tautomeric forms for the tetracyclic scaffold. Eltrombopag (entries 5 and 6) highlights how a different tautomeric form is processed by the ChEMBL pipeline in comparison to PubChem and canSAR pipelines. Without tautomer generation, the two different forms of Eltrombopag give two different outputs in ChEMBL. PubChem and canSAR pipelines generate the same canonical tautomer, regardless of the input structure itself.

**Table 4:**
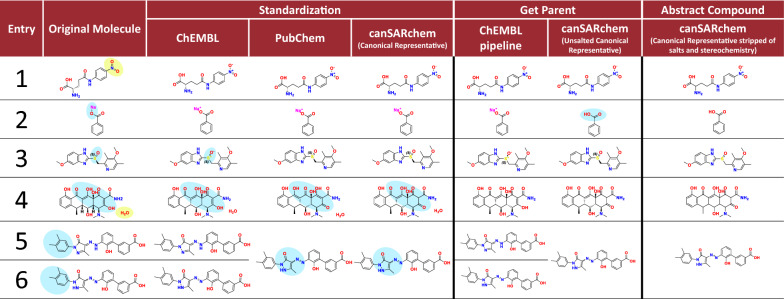
Standardized and abstract structure generated through canSAR, ChEMBL and PubChem pipelines

Examples show how the three examined pipelines deal with specific chemical issues: 1. Valence of Nitro group. 2. Metal bond disconnection 3. Sulphoxides standardization 4. Tautomer generation and salt strip 5 and 6. Double bond isomerism and tautomer generation. Compounds analysed have been chosen among those shown in the comparison tables in Bento’s paper [8]

We also analysed the similarities/discrepancies in the detection and rejection of problematic structures between the three pipelines. The ideal pipeline would be able to correct issues with structures and reduce the overall rejection rate. To this aim, we used the supplementary files provided in the ChEMBL [8] and PubChem [9] pipelines papers. A key issue faced by all three pipelines is the assumption that the structure originally registered into any of the source databases is correct and an accurate reflection of the publication/experiment. Our standardiser attempts to modify and correct structures wherever appropriate, i.e. in entry 1 in Table 4, the hypervalent nitro group is fixed during the standardisation. Table 5 highlights the rejection rates for canSAR and ChEMBL pipelines for 3 supplementary files from Bento and colleagues [8], namely a file from Sure ChEMBL with 52 k structures, a file from ChEMBL with about 300 k compounds and a file from ChEMBL literature containing 147 k compounds with 0 rejections. Overall the performances of canSARchem and ChEMBL pipeline were similar, with canSARchem being more inclusive with Sure ChEMBL and PubChem files but less inclusive with the ChEMBL literature file rejecting 3 compounds. Only 0.22% of structures were rejected by canSARchem, versus the 1.6% of ChEMBL in the case of the Sure ChEMBL file. Specifically, most of the errors were due to sanitization errors concerning valence and wedge errors. 97.5% of PubChem file compounds are accepted by canSARchem, with 3,261 more compounds compared to ChEMBL (96.41%). In this case, most of the errors causing rejection by canSARchem were due to problems with empty molblock. The rejection rate with ChEMBL literature was very similar.

**Table 5:** Detections of problematic structure (I)

	Sure_ChEMBL (SI1)	Pubchem (SI2)	ChEMBL literautre (SI3)
Structures #	52,074	297,864	147,008
ChEMBL pipeline errors (not uploaded structures)	849 (1.6%)	10,692 (3.59%)	0
ChEMBL uploaded structures	51,225 (98.37%)	287,172 (96.41%)	100%
canSAR pipeline errors (rejected structures)	114 (0.22%)	7431 (2.5%)	3 (0.002%)
SDF parsing errors	0	0	0
Sanitization errors	110	1540	2
Standardization errors	4	67	0
Empty molblock	0	5824	1
canSAR accepted structures	51,960 (99.78%)	290,433 (97.5%)	147,005 (99.99%)

Comparison with ChEMBL Checker on Supplementary files available in ChEMBL chemical standardization pipeline paper [8]. The canSAR pipeline is overall more inclusive with a lower percentage of rejected structures

Aiming at comparing the rejection rate with PubChem, we processed the Additional file 4 supplied in the PubChem standardization pipeline paper [9] through canSARchem. Specifically, this file contains substances that failed PubChem standardization but succeeded InChI normalization with the generation of the InChI string. The results, shown in Table 6, highlight the higher inclusiveness of canSARchem compared to PubChem. Indeed, only 76.07% of structures are rejected by canSARchem with about 90 k compounds that can still be uploaded (Table 6).

**Table 6:** Detections of problematic structure (II)

		PubChem Add_File_4
	Structures #	375,397
	PubChem pipeline errors (rejected compounds)	375,397 (100%)
ERRORS found PubChem Checker	Invalid isotope specifications	141
	Valence check	364,946 (97.22%)
	Identical charges on adjacent atoms or invalid valence after valence bond canonicalization	10,243
	Excess the limit of 999 explicit atoms	65
	canSAR pipeline errors (rejected compounds)	285,552 (76.07%)
	SDF parsing errors	0
	Sanitization errors	270,131 (71.96%)
	Standardization errors	2954 (0.78%)
	Empty molblock	12,467 (3.37%)
	canSAR accepted structures	89,845 (23.93%)

Comparison with PubChem Checker on Supplementary files available from PubChem chemical standardization pipeline paper [9]. Additional file 4 was used. The canSAR pipeline is overall more inclusive with a lower percentage of rejected structures but a superior performance in correcting wrong structure ahead of importing them in the database

To understand the reason for such differences in the percentage of rejection rate, we looked at some specific examples of compounds accepted by canSARchem. Table 7 includes three examples of compounds where canSARchem identified errors/inconsistencies and fixed them during the standardization and thus there were successfully accepted for registration. Entry 1 (compound 757 in Additional file 4 from PubChem) was successfully standardized and accepted by canSARchem although having an unusual valence on the sulphur atom. The detected error in PubChem concerns precisely the sulphur valence and the same issue (with a score of 6) is detected by the ChEMBL checker. Importantly, in the ChEMBL pipeline, the decision for rejecting or accepting structures is based on the score obtained in the Checker step. A score of 6 means that the compounds are loaded into the database but without a molfile, since the issue is considered to be relevant. Thus, the standardization process in ChEMBL is not diagnostic for wrong structures. This example illustrates a limitation of our pipeline which is not able to reject this compound despite the wrong valence. A similar case is shown in entry 2 (425,614), where PubChem detects the same error of the wrong valence for a sulphur atom. In this case, however, ChEMBL gives a score which is not related to the sulphur valence and the overall score would allow accepting the structure for database uploading. The examples in entries 3 and 4 represent the same compound (219,012) submitted to the pipelines as it was in the Additional file 4 (entry 3) and after canSAR standardization. canSAR and ChEMBL in this case outperform PubChem at fixing the hypervalent nitro group, generating an output structure that is perceived as correct by PubChem. Therefore, on the one hand, canSAR pipeline can be sometimes too inclusive in accepting compounds with a wrong valence, but, on the other hand, it is effective at fixing many compounds. Importantly, the issues around sulphur atom valence are not easy to solve, as demonstrated also by the different scores for a similar case given by ChEMBL to compounds from entries 1 and 2.

**Table 7:** Example of compounds rejected by PubChem pipeline

Entry	Input molecule	canSAR standardization (FICTS)	PubChem errors	ChEMBL standardization	ChEMBL errors
1			Detect illegal valence for element “S”		6, [‘InChI: accepted unusual valence(s)’], (2, ‘InChI: metal was disconnected’)
2			Detect illegal valence for element “S”		2, ‘InChI: Metal was disconnected’
3			Unable to fix pentavalent nitroso group		No issues detected
4			No issues detected		No issues detected

Examples of compounds rejected by the PubChem pipeline. All these compounds are valid according to the canSAR pipeline, which results to be more inclusive. Of note, inclusiveness of canSAR seems to be higher in edge cases with organometallic complexes where the valence is not easily perceived

### Statistical analysis of FDA-approved drugs

After processing all 3 million compounds through canSARchem, we investigated the degree to which FDA-approved drugs were affected for canonicalization and chirality and thus will be impacted by the amount of biological data that can be consolidated from different sources. We compared structures of FDA approved drugs (4094) against all other compounds in canSAR (3,153,790). To count compounds that were not registered as canonical forms, we compared the Standardized Compounds and the Canonical Representatives. To ensure structure uniqueness and to avoid different salt forms of the same drug counting as different compounds, we performed the comparison at the level of the Unsalted Standard Compound (which was generated ad hoc) and the Unsalted Canonical Tautomer. It is important to stress that there could be a small bias in this analysis as tautomers are only calculated for compounds with less than 100 atoms which are slightly less common among drugs (91.7%) than the rest of compounds (93.6%). H-fixed NonStandard InChIs of these forms were then compared showing that 9.7% (1,988,409) of all canSAR compounds were registered as non-canonical forms and underwent modifications during canonicalization. A much larger fraction (17.51%) of FDA-approved drugs were modified during canonicalization, showing a statistically significant enrichment (p value < 0.0001) in the chi-squared over all other compounds in canSAR. Correction of chirality, on the other hand, was similar between the two groups (44.78% for all canSAR compounds and 42.01% for FDA-approved drugs, Table 8). The enrichment of tautomeric forms among FDA-approved drugs highlights the importance of canonicalization for accurate data aggregation based on the chemical structure in public databases. To further assess the impact of the tautomerization, we looked for compounds families where the canSARchem pipeline allowed us to bring together data that would have otherwise been separated. We found 160 compounds families, containing at least one compound tagged as an FDA-approved drug, with at least two different tautomeric forms with registered bioactivity data. The above-mentioned tivozanib, pemetrexed and ganciclovir, together with valacyclovir, oxypurinol, dihydroxyacetone, malic acid and idelalisib are all examples of bioactivity and crystallography data linked to alternative tautomeric forms brought together through the canSARchem hierarchy.

**Table 8:** FDA-approved drugs enrichment

	All canSAR compounds	FDA-approved drugs	Chi-squared test
Total number of unique valid structures	1,988,409	2450	–
Compounds registered as non canonical forms	192,933 (9.7%)	429 (17.51%)	χ^2^ = 8,558,547,933 p-value < 0.0001
Chiral compounds	890,562 (44.78%)	1031 (42.01%)	Not enriched

FDA-approved drugs were compared to all compounds registered in canSAR for canonicalization and chirality enrichment. A significant p-value for compounds modified during canonicalization has been found through the Chi-squared test

## Conclusions

We have developed a chemical registration and standardization pipeline using KNIME and made it freely available. We used the pipeline to process > 3 M compounds available in canSAR, grouping them to enable users to rapidly identify alternative representations of any chemical compound and explore their bioactivities where appropriate. As exemplified in Figs. 3 and 4, the compound grouping created by canSARchem facilitates the identification of important bioactivity data and curation errors that may otherwise be missed. These hierarchies have been implemented in the canSAR website to facilitate their use. Despite its importance, canonical tautomerization continues to be a challenging, and not completely solved problem in cheminformatics that can affect some of the hierarchies. Our comparison between four different approaches demonstrates that the commercial software ChemAxon outperforms publicly available approaches and has therefore been chosen for the canSAR pipeline. However, the progress of public canonicalization approaches in RDKit is very encouraging and raise hopes of transitioning to open access canonicalization software in the near future. A thorough comparison between the canSAR, ChEMBL and PubChem pipelines demonstrates that all three pipelines show comparable performance in compound standardization. The main differences lie in the use of open-access software and canonicalization. ChEMBL uses fully open access software but does not perform canonicalization. PubChem includes a canonicalization step but under fully commercial software and many components of the pipeline are not available for users. canSARchem also performs canonicalization but uses mostly open-access software and the full pipeline is available under a Creative Commons licence but requires that users obtain their own ChemAxon license. canSARchem pipeline also has a slightly lower rejection rate compared to both PubChem and ChEMBL. Overall, canSARchem KNIME pipeline represents a solid compound registration and standardization approach, that generates a compound hierarchy that can help users identify valuable bioactivity data from related structures that otherwise could be missed. Given that FDA-approved drugs have shown a significant enrichment of canonicalization compared to other compounds in canSAR, our results also highlight the importance of canonicalizing in public medicinal chemistry databases and the urgent need for better canonicalization approaches.

## Supplementary Information

**Additional file 1: Figure S1. A** Files importer. sdf.gz files are imported through the *SDF Reader* node with their paths being read through the *List Files* node. **B** Structure Checker. Correct molecules are imported in KNIME through the *SDF* *Reader* node and after kekulization (by means of the *Dearomatizer* node) are converted into RDKit mol format and partially sanitised using the *RDKit from Molecule* node. As a last sanity check, compounds with empty mol block are removed through the *RDKit Substructure Filter* node and are ready for the standardization step. All wrong compounds identified through these steps go to the *Errors_No_Structures* metanode to be written in the *Errors* or *No_Structures* folders. **Figure S2.** Examples of differences in neutralization between MolVS, ChemAxon and RDKit. In BindingDB, 11,284 molecules were charged after RDKit standardization. From them, 5212 molecules were still charged after Neutralization performed through ChemAxon Standardizer, including inorganic salts and charged functional groups (e.g., nitro). In contrast, about 200 additional compounds (5063 charged molecules after RDKit neutralization) were neutralized by the RDKit neutralization function in comparison to ChemAxon, i.e., entries 1 and 2 (rdMolStandardize.Uncharger module). Examples below illustrate these differences. Users should be mindful about the differences, particularly when using the fully open access version of our pipeline, canSARchemRDKit, that may neutralize some additional compounds. **Figure S3.** Examples of metal bonds. Entry 1 represents a case where the metal bond was not processed during RDKit standardization and was disconnected through the subsequent extra step. Entry 2 represents a case where RDKit standardization efficiently dealt with metal bond. **Figure S4.** Standardizer. Compounds standardization is performed through RDKit. Exceptions are dealt with separately using an additional neutralization step as well as an extra step to break metal bonds, which are not recognized by RDKit. **Figure S5.** Generation of canonical representatives. ChemAxon/Infocom Tautomers node is used to generate the canonical tautomers with the protection of stereochemistry and double bond annotations. **Figure S6.** Salt Strip. Sequential steps for stripping salts and solvents from compounds and generating the unsalted canonical representatives. **Figure S7.** Generation of abstract compound. Abstract compound is obtained through clearing stereoisomerism, double bond isomerism and isotopes annotations aiming to group compounds into families. **Table S1.** Comparison of ChemAxon and RDKit canonicalization. ChemAxon and RDKit canonicalization have been executed protecting double-bond isomerism and stereochemistry. Entry 1 and 4 show examples of ineffective protection of double-bond isomerism by RDKit tool. In entry 2 and 3, instead, the stereochemistry label is lost in the RDKit output. In all cases, ChemAxon output was the same as the input molecule evidencing its higher reliability at protecting both stereo and double-bond isomerism.

## Data Availability

The datasets supporting the conclusions of this article are available in ChEMBL (https://www.ebi.ac.uk/chembl/), PubChem (https://pubchem.ncbi.nlm.nih.gov/) and PDB (https://www.rcsb.org/).
