# Supplementary material for: canSAR chemistry registration and standardization pipeline
Source: J Cheminform. 2022 May 28;14:28. doi: 10.1186/s13321-022-00606-7 (PMC9148294; doi:10.1186/s13321-022-00606-7)
Supplement: Supplementary file 1 — Additional file 1: Figure S1. A Files importer. sdf.gz files are imported through the SDF Reader node with their paths being read through the List Files node. B Structure Checker. Correct molecules are imported in KNIME through the SDF Reader node and after kekulization (by means of the Dearomatizer node) are converted into RDKit mol format and partially sanitised using the RDKit from Molecule node. As a last sanity check, compounds with empty mol block are removed through the RDKit Substructure Filter node and are ready for the standardization step. All wrong compounds identified through these steps go to the Errors_No_Structures metanode to be written in the Errors or No_Structures folders. Figure S2. Examples of differences in neutralization between MolVS, ChemAxon and RDKit. In BindingDB, 11,284 molecules were charged after RDKit standardization. From them, 5212 molecules were still charged after Neutralization performed through ChemAxon Standardizer, including inorganic salts and charged functional groups (e.g., nitro). In contrast, about 200 additional compounds (5063 charged molecules after RDKit neutralization) were neutralized by the RDKit neutralization function in comparison to ChemAxon, i.e., entries 1 and 2 (rdMolStandardize.Uncharger module). Examples below illustrate these differences. Users should be mindful about the differences, particularly when using the fully open access version of our pipeline, canSARchemRDKit, that may neutralize some additional compounds. Figure S3. Examples of metal bonds. Entry 1 represents a case where the metal bond was not processed during RDKit standardization and was disconnected through the subsequent extra step. Entry 2 represents a case where RDKit standardization efficiently dealt with metal bond. Figure S4. Standardizer. Compounds standardization is performed through RDKit. Exceptions are dealt with separately using an additional neutralization step as well as an extra step to break metal bonds, which are not reco [file 13321_2022_606_MOESM1_ESM.docx]

**Figure S1.** **A. Files importer.** sdf.gz files are imported through the *SDF Reader* node with their paths being read through the *List Files* node. **B**. **Structure Checker.** Correct molecules are imported in KNIME through the SDF Reader node and after kekulization (by means of the *Dearomatizer* node) are converted into RDKit mol format and partially sanitised using the *RDKit from Molecule* node. As a last sanity check, compounds with empty mol block are removed through the *RDKit Substructure Filter* node and are ready for the standardization step. All wrong compounds identified through these steps go to the *Errors_No_Structures* metanode to be written in the *Errors* or *No_Structures* folders.


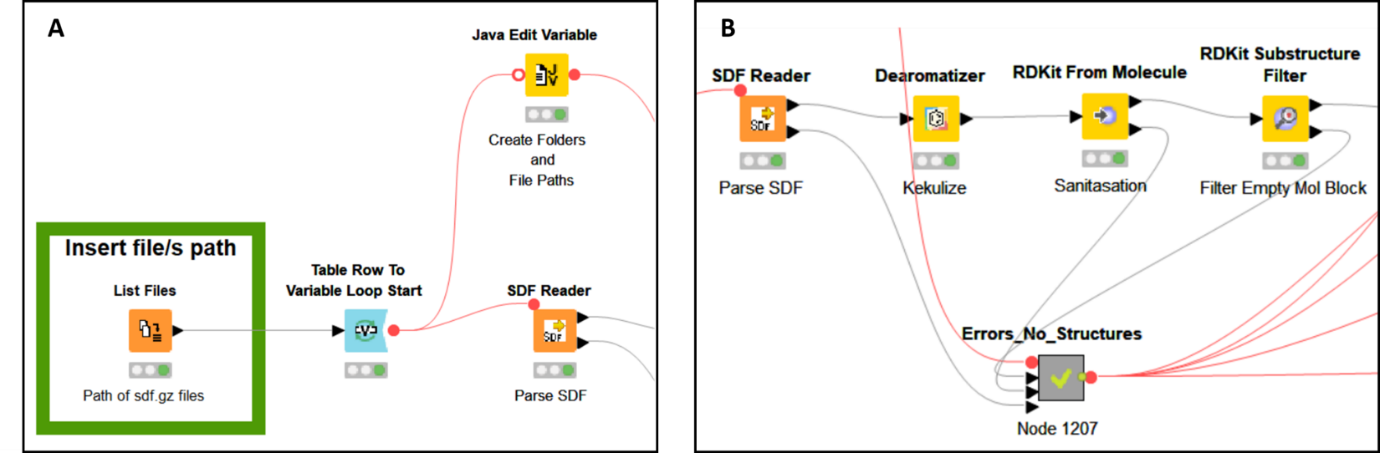


**Figure S2. Examples of differences in neutralization between MolVS, ChemAxon and RDKit.** In BindingDB, 11,284 molecules were charged after RDKit standardization. From them, 5,212 molecules were still charged after Neutralization performed through ChemAxon Standardizer, including inorganic salts and charged functional groups (e.g. nitro). In contrast, about 200 additional compounds (5063 charged molecules after RDKit neutralization) were neutralized by the RDKit neutralization function in comparison to ChemAxon, i.e. entries 1 and 2 (rdMolStandardize.Uncharger module). Examples below illustrate these differences. Users should be mindful about the differences, particularly when using the fully open access version of our pipeline, canSARchemRDKit, that may neutralize some additional compounds.


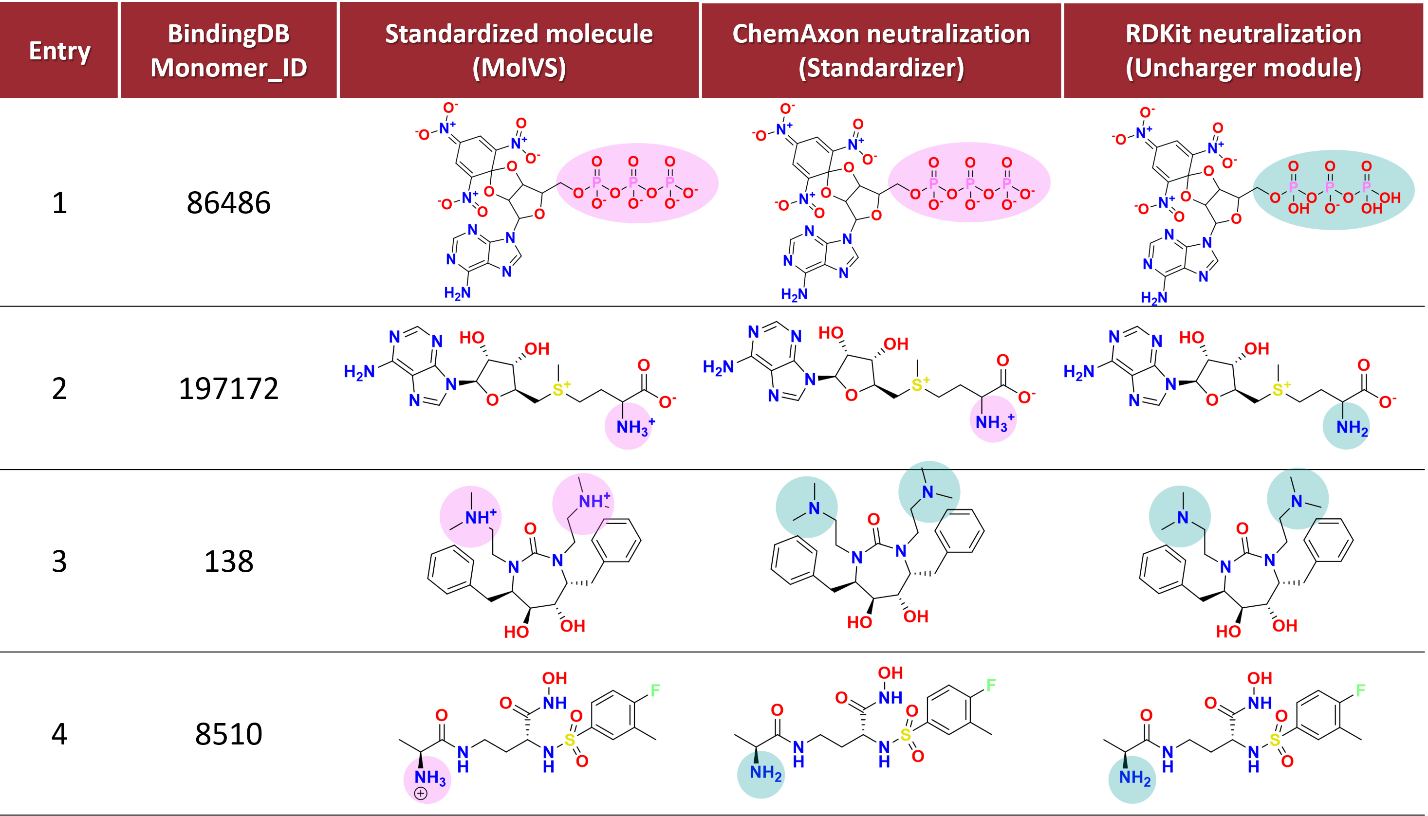


**Figure S3.** Examples of metal bonds. Entry 1 represents a case where the metal bond was not processed during RDKit standardization and was disconnected through the subsequent extra step. Entry 2 represents a case where RDKit standardization efficiently dealt with metal bond.


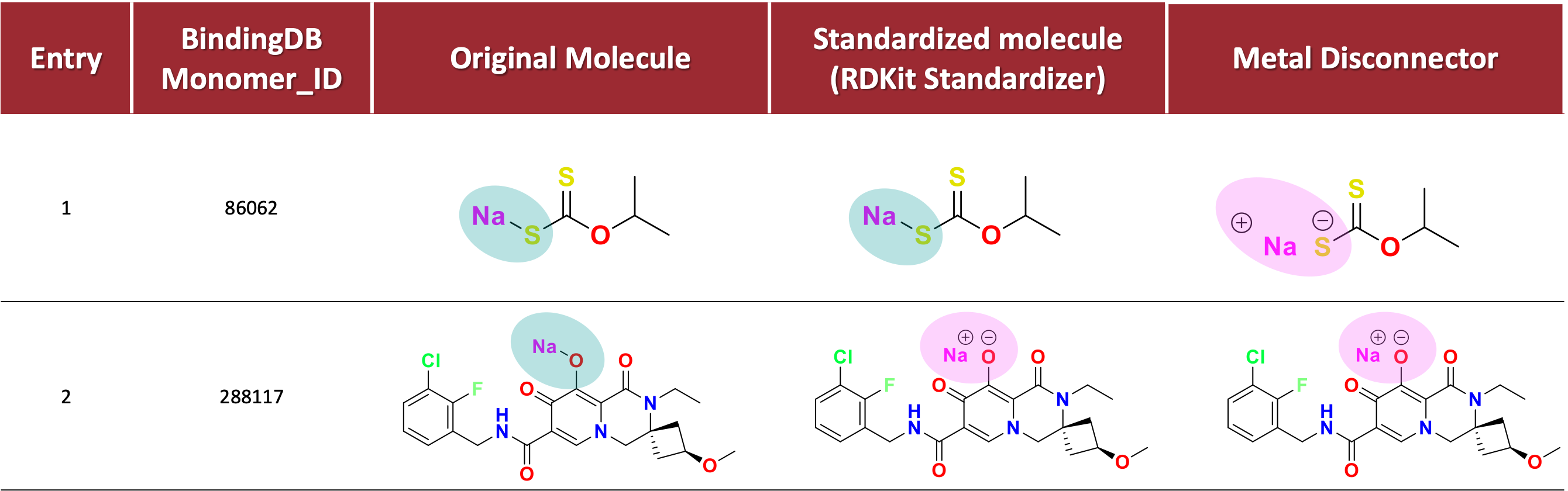


**Figure S4. Standardizer.** Compounds standardization is performed through RDKit. Exceptions are dealt with separately using an additional neutralization step as well as an extra step to break metal bonds, which are not recognized by RDKit.

**
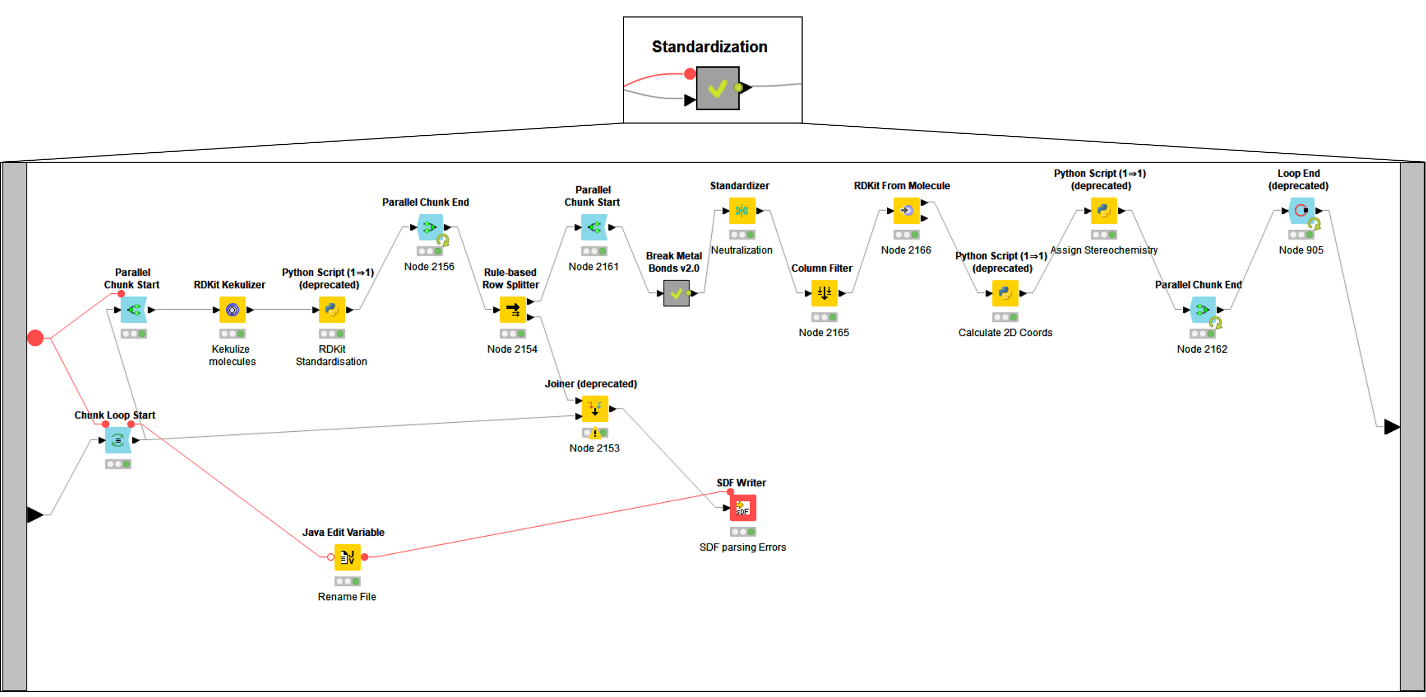
**

**Figure S5. Generation of canonical representatives.** ChemAxon/Infocom Tautomers node is used to generate the canonical tautomers with the protection of stereochemistry and double bond annotations.


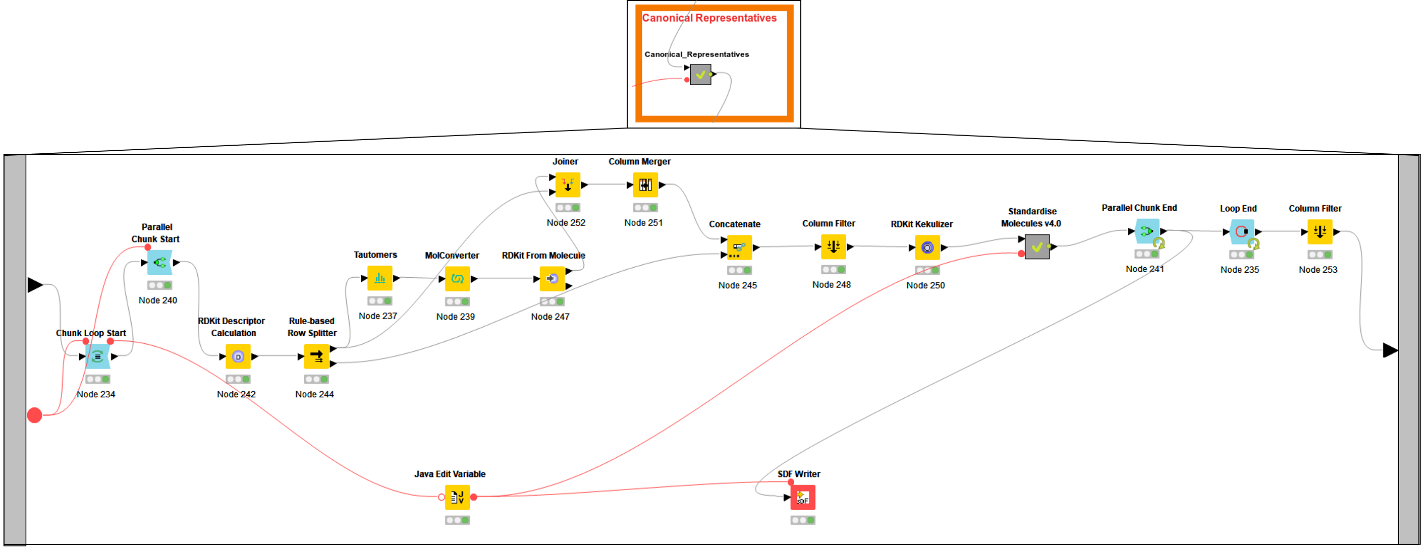


**Figure S6.** **Salt Strip**. Sequential steps for stripping salts and solvents from compounds and generating the unsalted canonical representatives.


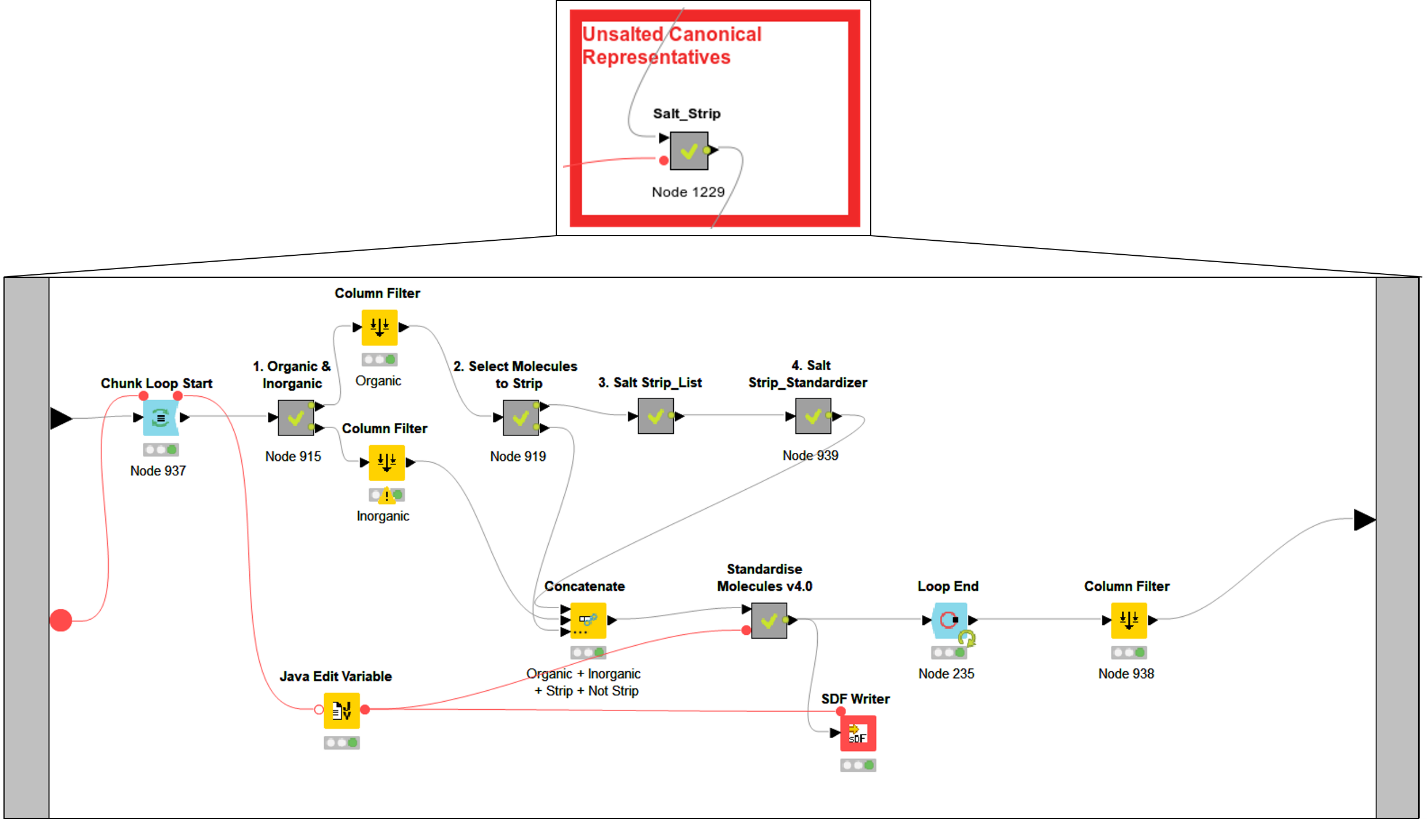


## Figure S7. Generation of abstract compound. Abstract compound is obtained through clearing stereoisomerism, double bond isomerism and isotopes annotations aiming to group compounds into families.

##
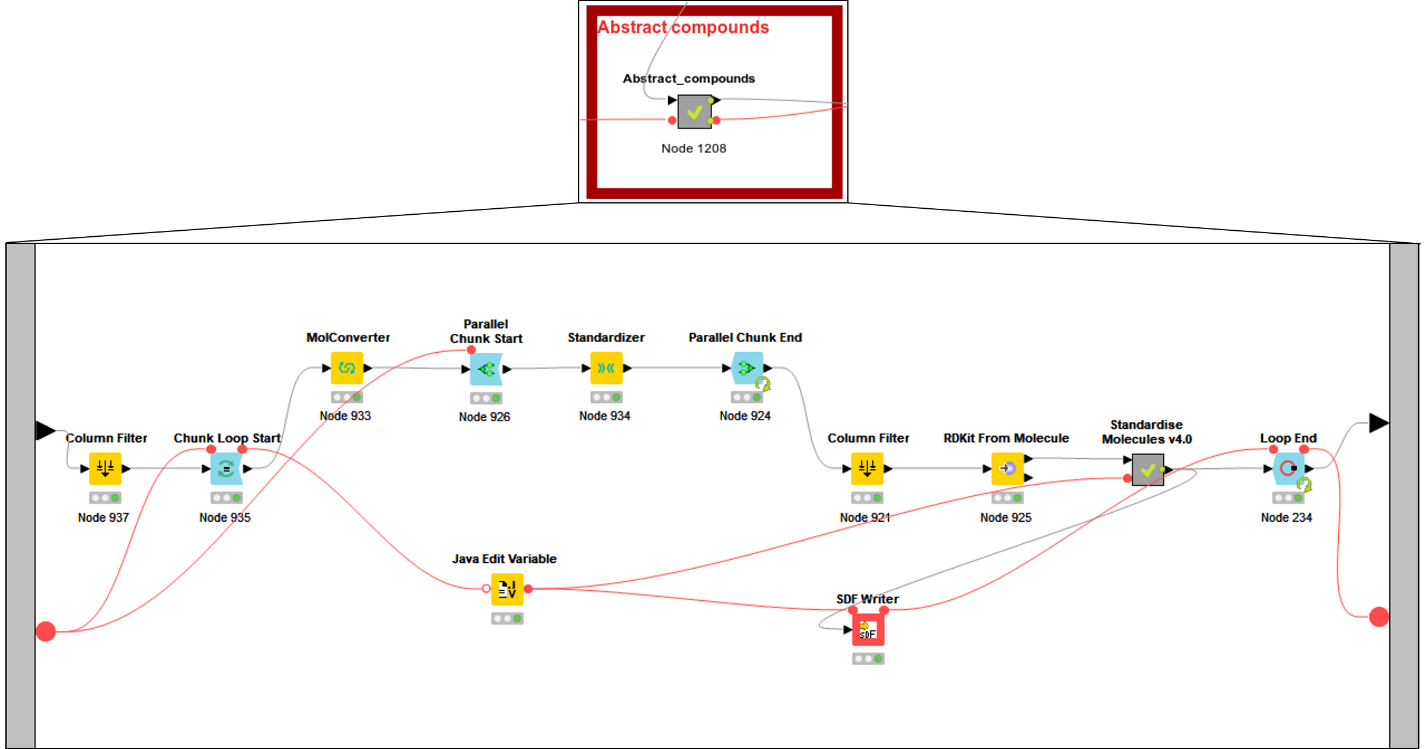


**Table S1. Comparison of ChemAxon and RDKit canonicalization**

ChemAxon and RDKit canonicalization have been executed protecting double-bond isomerism and stereochemistry. Entry 1 and 4 show examples of ineffective protection of double-bond isomerism by RDKit tool. In entry 2 and 3, instead, the stereochemistry label is lost in the RDKit output. In all cases, ChemAxon output was the same as the input molecule evidencing its higher reliability at protecting both stereo and double-bond isomerism.

**
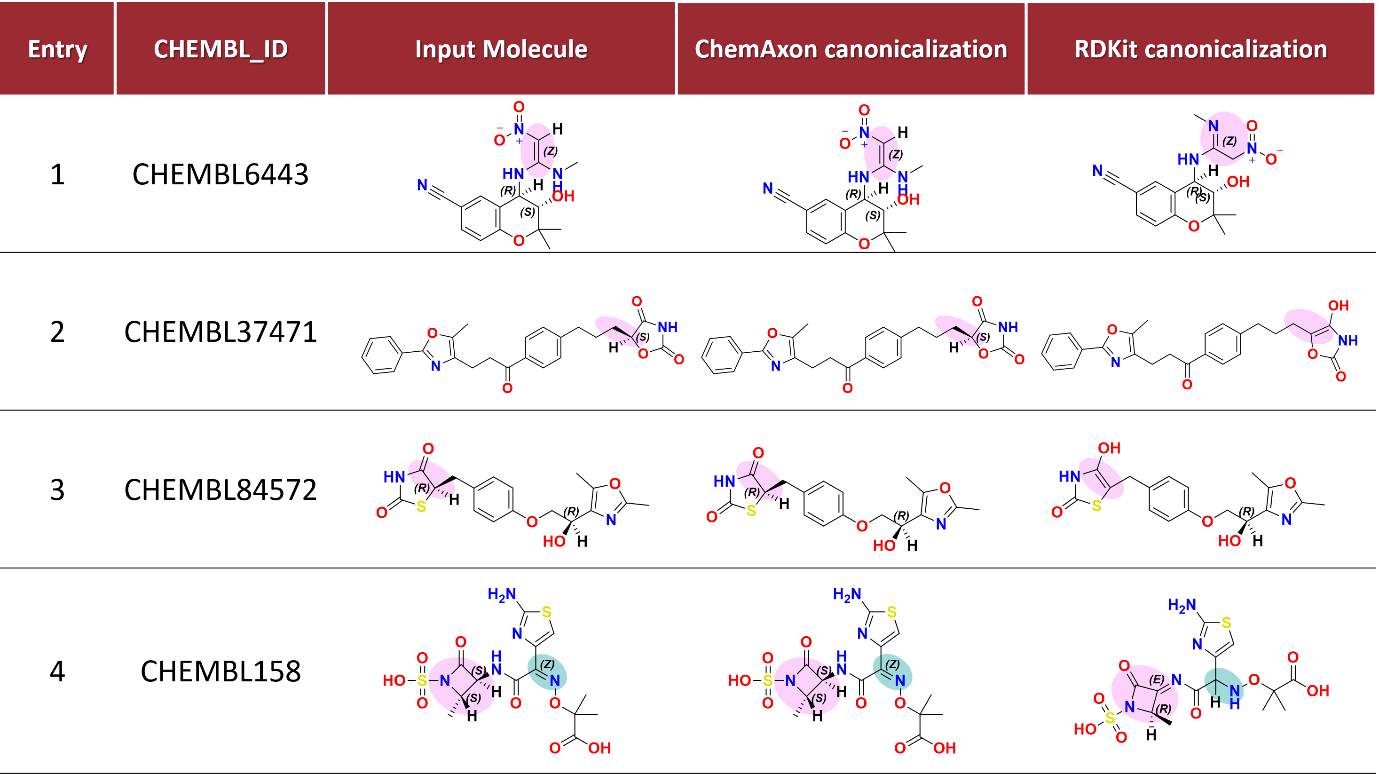
**
